# Fibroblast‐derived miR‐425‐5p alleviates cardiac remodelling in heart failure via inhibiting the TGF‐β1/Smad signalling

**DOI:** 10.1111/jcmm.70199

**Published:** 2024-11-11

**Authors:** Haijia Zhou, Pengyun Liu, Xuelin Guo, Wei Fang, Chan Wu, Mingming Zhang, Zhaole Ji

**Affiliations:** ^1^ Department of Cardiology Tangdu Hospital, Second Affiliated Hospital of Air Force Medical University Xi'an China

**Keywords:** cardiac remodelling, fibroblast, heart failure, miR‐425‐5p, TGF‐β1/Smad, transverse aortic constriction

## Abstract

The pathological activation of cardiac fibroblasts (CFs) plays a crucial role in the development of pressure overload‐induced cardiac remodelling and subsequent heart failure (HF). Growing evidence demonstrates that multiple microRNAs (miRNAs) are abnormally expressed in the pathophysiologic process of cardiovascular diseases, with miR‐425 recently reported to be potentially involved in HF. In this study, we aimed to investigate the effects of fibroblast‐derived miR‐425‐5p in pressure overload‐induced HF and explore the underlying mechanisms. C57BL/6 mice were injected with a recombinant adeno‐associated virus specifically designed to overexpress miR‐425‐5p in CFs, followed by transverse aortic constriction (TAC) surgery. Neonatal mouse CFs (NMCFs) were transfected with miR‐425‐5p mimics and subsequently stimulated with angiotensin II (Ang II). We found that miR‐425‐5p levels were significantly downregulated in HF mice and Ang II‐treated NMCFs. Notably, fibroblast‐specific overexpression of miR‐425‐5p markedly inhibited the proliferation and differentiation of CFs, thereby alleviating myocardial fibrosis, cardiac hypertrophy and systolic dysfunction. Mechanistically, the cardioprotective actions of miR‐425‐5p may be achieved by targeting the TGF‐β1/Smad signalling. Interestingly, miR‐425‐5p mimics‐treated CFs could also indirectly affect cardiomyocyte hypertrophy in this course. Together, our findings suggest that fibroblast‐derived miR‐425‐5p mitigates TAC‐induced HF, highlighting miR‐425‐5p as a potential diagnostic and therapeutic target for treating HF patients.

## 1 INTRODUCTOIN

Heart failure (HF) is the end stage of various cardiovascular diseases (CVDs), including coronary heart disease, hypertension, valvular heart disease and cardiomyopathy, posing a growing socioeconomic burden worldwide.^1^, ^2^ Among the multiple pathogenic factors of HF, pressure overload has garnered increasing attention. Although a moderate increase in cardiac load initially has a compensatory effect, prolonged and sustained pressure overload would eventually induce pathological cardiac remodelling and decompensated cardiac function, leading to chronic HF and, in some cases, sudden death.^3^, ^4^

Recently, accumulating evidence indicates that reversing or retarding the progression of cardiac remodelling has become an important strategy for reducing hospitalization rates and improving long‐term survival in patients with HF.^5^ Excessive myocardial fibrosis and irreversible myocardial hypertrophy caused by chronic stress or diseases are hallmark clinical manifestations and key pathophysiological processes of adverse remodelling.^6^ Significantly, disordered activation of cardiac fibroblasts and excessive deposition of extracellular matrix reduce ventricular wall compliance, aggravate cardiac dysfunction, and ultimately accelerate the progression of HF.^7^, ^8^ Hence, identifying potential therapeutic targets and elucidating the underlying mechanisms of cardiac remodelling are of utmost importance for treating HF.

MicroRNAs (miRNAs), a class of endogenous small non‐coding RNAs, regulate target mRNA by disrupting its stability and inhibiting its translation.^9^ Numerous studies have suggested that miRNAs could serve as biomarkers for the diagnosis or treatment of certain diseases due to their roles in modulating various physiological and pathological processes.^10^, ^11^ Currently, the involvement of miR‐425 has been uncovered in several CVDs, such as myocardial infarction, viral myocarditis, doxorubicin‐induced cardiotoxicity, and HF.^12^, ^13^, ^14^, ^15^ Of note, overexpression of miR‐425 has been reported to exert profound cardioprotective effects in these conditions. Previous studies have also found that miR‐425 level in the plasma exosomes can predict cardiac fibrosis, and miR‐425‐5p is negatively correlated with atrial fibrosis and promotes atrial remodelling.^16^, ^17^ However, whether miR‐425‐5p in heart tissue could exhibit antifibrotic action to attenuate cardiac remodelling and subsequently HF remains to be explored. Moreover, given that the dysregulation of TGF‐β1/Smad pathway is a crucial pathogenic mechanism of myocardial fibrosis,^18^ and that miR‐425 could modulate the TGF‐β family pathway,^19^, ^20^ it is intriguing to investigate the regulatory role of miR‐425‐5p on the TGF‐β1/Smad pathway in this process.

Here, this study aimed to investigate the effects of miR‐425‐5p on pressure overload‐induced HF and elucidate its underlying mechanisms. Our experiments initially observed that miR‐425‐5p levels were downregulated in both heart tissue and plasma of HF mice, and then demonstrated that miR‐425‐5p acts as an upstream regulator of the TGF‐β1/Smad signalling in inhibiting CFs activation, thereby mitigating TAC‐induced over‐activated fibrotic and hypertrophic response. These findings suggest that specific overexpression of miR‐425‐5p in CFs plays a pivotal role in alleviating pathological myocardial remodelling and cardiac dysfunction, providing a novel paradigm for the treatment of chronic HF.

## 2 MATERIALS AND METHODS

### 2.1 Animals

Male C57BL/6 mice aged 8–12 weeks and 1–3 days were purchased from the Laboratory Animal Center of Air Force Medical University (Shaanxi, China). All experiments involving animals conformed to the Guide for the Care and Use of Laboratory Animals published by the US National Institutes of Health (NIH Publication No. 85–23, revised 1985), and were approved by the Ethic Committee on Animal Care of Air Force Medical University (Approval Document No. IACUC‐20230220).

### 2.2 Reagents

Angiotensin II (Ang II) was purchased from Solarbio Biotechnology (Beijing, China). SRI‐011381, an activator of TGF‐β signalling pathway, was purchased from MedChem Express (Monmouth Junction, NJ, USA). Primary antibodies against transforming growth factor beta‐1 (TGF‐β1, ab92486), alpha‐smooth muscle actin (α‐SMA, ab7817), Ki67 (ab16667), α‐actinin (ab108198) and Histone H3 (ab1791) were obtained from Abcam (Cambridge, MA, USA). Primary antibodies against p‐Smad2 (Ser465/Ser467, #18338), t‐Smad2 (#5339), p‐Smad3 (Ser423/Ser425, #9520), t‐Smad3 (#9523) and Smad4 (#46535) were purchased from Cell Signalling Technology (Boston, MA, USA). Primary antibodies against BrdU (66241‐1‐Ig), vimentin (10366‐1‐AP), proliferating cell nuclear antigen (PCNA, 10205‐2‐AP) and glyceraldehyde‐3‐phosphate dehydrogenase (GAPDH, 60004‐1‐Ig) were purchased from Proteintech (Chicago, IL, USA). The goat anti‐rabbit and goat anti‐mouse secondary antibodies were purchased from the Zhongshan Company (Beijing, China). miR‐425‐5p mimics (mimic‐miR), miRNA mimic negative control (mimic‐NC), miR‐425‐5p inhibitors (inhibitor‐miR) and miRNA inhibitor NC (inhibitor‐NC) were designed and synthesized by RiboBio (Guangzhou, China). The primers of miR‐425‐5p and U6 were also provided by RiboBio (Guangzhou, China). Real‐time PCR primers of atrial natriuretic peptide (ANP), brain natriuretic peptide (BNP), fibronectin (FN), connective tissue growth factor (CTGF), Collagen 1a1 (Col1a1), Collagen 3a1 (Col3a1) and GAPDH were designed and synthesized by Sangon Biotech (Shanghai, China).

### 2.3 Transverse aortic constriction (TAC) surgery

Mouse model of pressure overload‐induced HF was performed by TAC surgery as previously described.^21^ In brief, after being anaesthetised with 2% isoflurane, mice were endotracheally intubated and mechanically ventilated using Minivent 845 (Hugo Sachs Electronic, Germany) at a tidal volume of 0.15 mL. The transverse aorta was fully exposed through median thoracotomy. Subsequently, the aortic arch was constricted with a 27‐gauge needle using 7–0 silk suture ligature between the innominate and left common carotid arteries, and the chest wall was then closed. Sham‐operated mice underwent the aforementioned procedures without the ligation of the aortic arch. Eight weeks after surgery, echocardiography was performed before the mice were sacrificed, and the plasma and organs were harvested for subsequent experiments.

### 2.4 Construction of recombinant adeno‐associated virus (rAAV)

The rAAV system (type 9) was used to manipulate the expression of miR‐425‐5p in vivo. We employed a periostin (POSTN) promoter to exclusively express miR‐425‐5p in CFs. The rAAVs were packaged by triple plasmid co‐transfection, as described previously.^22^

### 2.5 In vivo experiment

①To explore the effects of miR‐425‐5p on TAC‐induced HF. Eighty C57BL/6 mice were randomly assigned to the following four groups: Sham+rAAV9‐con group, Sham+rAAV9‐miR group, TAC + rAAV9‐con group and TAC + rAAV9‐miR group, with 20 mice in each group. Mice in the Sham+rAAV9‐con group and Sham+rAAV9‐miR group underwent the sham operation, while mice in the TAC + rAAV9‐con group and TAC + rAAV9‐miR group underwent the TAC operation. For overexpression of miR‐425‐5p in vivo, the mice were administered rAAV9‐POSTN‐miR‐425‐5p (100 μL/mouse) or an equal dose of rAAV9‐POSTN‐con by tail vein injection 2 weeks before sham or TAC surgery. Protocol of in vivo experiments ① was shown in Figure S1.

②To explore the molecular mechanism of miR‐425‐5p on TAC‐induced HF. Eighty C57BL/6 mice were randomly assigned to the following four groups: Sham+rAAV9‐con group, TAC + rAAV9‐con group, TAC + rAAV9‐miR group and TAC + rAAV9‐miR + SRI group, with 20 mice in each group. Mice in the Sham+rAAV9‐con group underwent the sham operation, while mice in the TAC + rAAV9‐con group, TAC + rAAV9‐miR group and TAC + rAAV9‐miR + SRI group underwent the TAC operation. rAAV9‐POSTN‐miR‐425‐5p or rAAV9‐POSTN‐con were administrated as ①, while SRI‐011381 (30 mg/kg) was intraperitoneally injected every 5 days for 8 weeks. Protocol of in vivo experiments ② was shown in Figure S2.

### 2.6 Isolation of neonatal mouse cardiomyocytes (NMCMs) and cardiac fibroblasts (NMCFs)

NMCMs and NMCFs were isolated from 1 to 3‐day‐old C57BL/6 mice as described previously.^23^ Left ventricles were minced and digested with 0.1% collagenase II and 0.08% trypsin at 37°C. After digestion, all cells were harvested and then pre‐plated for 1.5 h. Cardiac fibroblasts (CFs) were isolated from cardiomyocytes (CMs) by differential adhesion time, and the adherent cells were CFs that positively expressed vimentin, while the suspended cells were CMs that positively expressed α‐actinin. Then, cells were maintained in DMEM supplemented 10% fetal bovine serum (FBS), and incubated at 37°C in humidified chamber.

### 2.7 In vitro experiment

A pressure‐overload in vitro model was mimicked by incubating with Ang II (1 μM) for 24 h as previously described.^24^ While the control (Con) cells were cultured in the normal DMEM for 24 h. ①NMCFs were randomly assigned to the following four groups: Con+mimic‐NC group, Con+mimic‐miR group, Ang II + mimic‐NC group and Ang II + mimic‐miR group. ②NMCFs were randomly assigned to the following four groups: Con+mimic‐NC group, Ang II + mimic‐NC group, Ang II + mimic‐miR group and Ang II + mimic‐miR + SRI group. To overexpress miR‐425‐5p in vitro, NMCFs were transfected with miR‐425‐5p mimics (100 nM) or mimic NC (100 nM) for 48 h using Lipofectamine 3000 (Invitrogen, CA, USA) according to the manufacturer's instructions. To activate the TGF‐β signalling pathway, SRI‐011381 was added to the culture medium at a dose of 10 μM. Cells were then treated with normal DMEM, Ang II or SRI‐011381 for 24 h after transfection for subsequent experiments.

### 2.8 Indirect co‐culture model

NMCFs seeded in culture dish were transfected with miR‐425‐5p mimics or mimic NC for 48 h and then stimulated with Ang II (1 μM) for another 24 h to induce a fibrotic phenotype of CFs. The conditioned supernatants from each group were collected and centrifuged at 1500 rpm for 5 min. Isolated NMCMs were cultured in the conditioned supernatants for up to 48 h.

### 2.9 Echocardiography

Cardiac systolic function was assessed by transthoracic echocardiography. A two‐dimensional parasternal long‐axis view was performed using a VisualSonics Vevo 770 ultrasound system (VisualSonics, Toronto, Ontario, Canada). And an M‐mode echocardiography was recorded at the level of the papillary muscles. Left ventricular ejection fraction (LVEF) and left ventricular fractional shortening (LVFS) were calculated according to the standard formulae.

### 2.10 Histological measurement

Eight weeks after the sham or TAC operation, the mice were weighed and euthanized, at the same time, their hearts and lungs were quickly harvested. The heart weight (HW), lung weight (LW) and body weight (BW) were measured in order to calculate the HW/BW and LW/BW ratios. Hearts sections were stained with Masson's trichrome and picric acid sirius red to evaluate myocardial fibrosis and collagen deposition, respectively. Wheat germ agglutinin (WGA) staining was used to measure mean cross‐sectional area (CSA) of cardiomyocyte as described previously.^25^

### 2.11 Quantitative real‐time PCR (qRT‐PCR)

Total RNA was extracted from heart tissues or cultured cells with TRIzol Reagent (Sigma‐Aldrich, St. Louis, MO, USA) and reverse transcribed into cDNA using the QuantiTect Reverse Transcription Kit (QIAGEN, Germany), followed by qRT‐PCR with SYBR Premix Ex TaqTM kit (Takara, Beijing, China). The 2^−ΔΔCT^ method was used to analyse the data, and the results were presented as relative value to the sham or con group. GAPDH was detected as the internal standards for mRNA. The primers adopted in this process were listed in Table 1.

**TABLE 1:** The sequences of indicated primers used in this study.

Gene	Forward(5′‐3′)	Reverse(5′‐3′)
ANP	AGGCAGTCGATTCTGCTTGA	CGTGATAGATGAAGGCAGGAAG
BNP	TAGCCAGTCTCCAGAGCAATTC	TTGGTCCTTCAAGAGCTGTCTC
CTGF	GCTGCCTACCGACTGGAAGAC	CCTAATGGCTTCCACCCTCTTC
FN	CCGGTGGCTGTCAGTCAGA	CCGTTCCCACTGCTGATTTATC
Col1a1	CCGAGGTATGCTTGATCT	GACAGTCCAGTTCTTCATTG
Col3a1	ATGGTGGTTTTCAGTTCAGC	GCCTTGAATTCGCCTTCATT
GAPDH	GGTTGTCTCCTGCGACTTCA	GGTGGTCCAGGGTTTCTTACTC

### 2.12 miRNA qPCR assay

The miR‐425‐5p from mouse heart and plasma were extracted using the miRNeasy Mini Kit and miRNeasy Serum/Plasma Kit (QIAGEN, Germany). The miRcute Plus miRNA qPCR Kit (TIANGEN, Beijing, China) was used to measure the miR‐425‐5p levels. The forward primers of mmu‐miR‐425‐5p and U6 small nuclear RNA were 5′‐AAUGACACGAUCACUCCCGUUGA‐3′ and 5′‐CAAATTCGTGAAGCGTTCCATAT‐3′. qPCR was conducted through a universal reverse primer (TIANGEN, Beijing, China).

### 2.13 Dual‐luciferase reporter assay

HEK293 cells were obtained from American Type Culture Collection (ATCC) (Manassas, VA) and cultured in DMEM supplemented with 10% FBS. The wide type (WT) and mutation (Mut) 3′‐UTR of TGF‐β1 genes were cloned into the pmirGLO vector (Promega, WI, USA). Then, HEK293 cells were transfected with 400 ng of pMIR‐TGF‐β1 WT or pMIR‐TGF‐β1 Mut vector, respectively. Meanwhile, miR‐425‐5p mimics or mimic NC was co‐transfected with those reporter plasmids at a final concentration of 100 nM. After 48 h, a dual‐luciferase reporter assay system (Promega, WI, USA) was used to measure the luciferase activity.

### 2.14 Immunofluorescence (IF) staining

Paraffin sections and paraformaldehyde‐fixed cells were incubated with bovine serum albumin (BSA) at room temperature. Anti‐α‐SMA, anti‐PCNA, anti‐BrdU, anti‐vimentin and anti‐α‐actinin antibodies (1:300 ~ 1:500) were used for IF staining overnight at 4°C, followed by incubations with corresponding secondary antibodies (1:100). 4, 6‐diamidino‐2‐phenylindole (DAPI) or Hoechst 33342 reagents were used to stain the nucleus. The relative area of NMCMs and quantitative analysis of α‐SMA expression, PCNA^+^ nuclei and BrdU^+^ nuclei in heart tissues or NMCFs were performed using ImageJ software (Bethesda, MD, USA).

### 2.15 Western blotting

Total heart and cytoplasmic proteins were extracted in RIPA lysis buffer. Nuclear extracts were prepared using a nuclear extraction kit (Sigma‐Aldrich, MO, USA). The protein concentrations were measured using the BCA Protein Assay Kit. Protein samples were separated by SDS‐PAGE and then transferred to polyvinylidene difluoride (PVDF) membranes. The blots were blocked with 5% skim milk for 2 h and then incubated with primary antibodies (1:1000) at 4°C overnight. After being rinsed with TBST, the membranes were incubated with HRP‐conjugated secondary antibodies (1:5000) for 1 h. Finally, the bands were visualized using a chemiluminescence system (Bio‐Rad Laboratories, Hercules, CA, USA), and analysed using ImageJ software. Quantitative expressions of α‐SMA, Ki67 and TGF‐β1 were normalized against GAPDH, and p‐Smads were normalized to t‐Smads, while quantitative expression of Smad4 was normalized against Histone H3.

### 2.16 Statistical analysis

Statistical analysis was performed using GraphPad Prism 9.0 (GraphPad Software, Inc., San Diego, CA, USA) and presented as the mean ± standard error of mean (SEM). Differences between two groups were assessed by unpaired *t* test with Welch's correction. While differences between multiple groups were analysed with one‐way ANOVA followed by the Bonferroni correction for post hoc *t* test. *p* < 0.05 was considered statistically significant.

## 3 RESULTS

### 3.1 miR‐425‐5p levels were downregulated in HF mice and Ang II‐treated NMCFs

**FIGURE 1:**
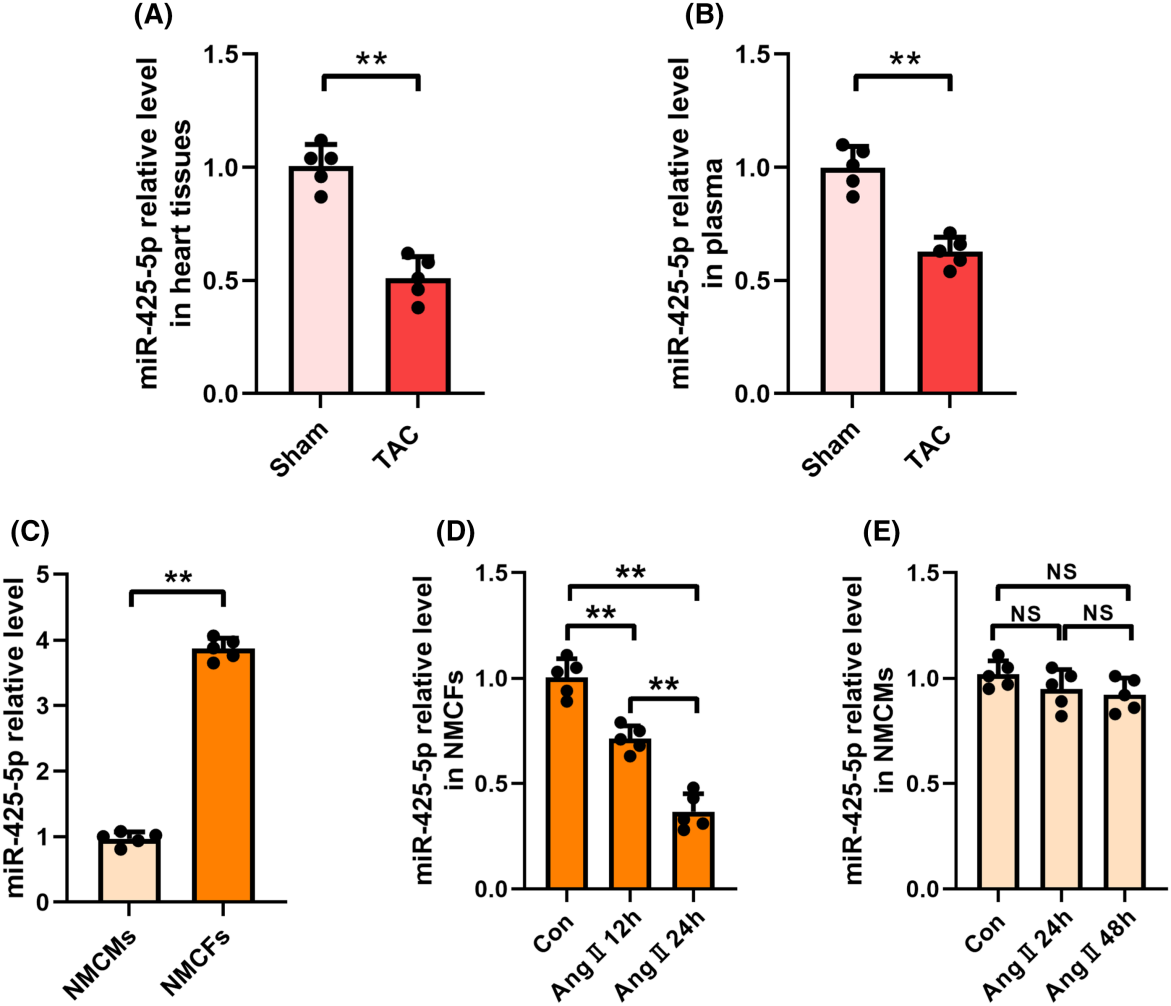
miR‐425‐5p levels were downregulated in HF mice and Ang II‐treated NMCFs. (A) qPCR analysis of miR‐425‐5p level in the heart tissues at 8 weeks after sham or TAC surgery (*n* = 5). (B) qPCR analysis of miR‐425‐5p level in the plasma at 8 weeks after sham or TAC surgery (*n* = 5). (C) qPCR analysis of miR‐425‐5p level in NMCMs and NMCFs (*n* = 5). (D) qPCR analysis of miR‐425‐5p level in NMCFs after incubation with Ang II for 12 and 24 h (*n* = 5). (E) qPCR analysis of miR‐425‐5p level in NMCMs after incubation with Ang II for 24 and 48 h (*n* = 5). HF, heart failure; NMCMs, neonatal mouse cardiomyocytes; NMCFs, neonatal mouse cardiac fibroblasts. Data were presented as mean ± SEM. Statistical significance was assessed by one‐way ANOVA. **p < 0.01; NS, no significant.

To investigate whether miR‐425‐5p is involved in the pathophysiological processes related to HF, we evaluated miR‐425‐5p levels in heart tissues and plasma from sham‐ or TAC‐operated mice. qPCR assays showed that miR‐425‐5p levels were significantly downregulated in both the whole heart tissue and plasma of TAC‐caused HF mice (Figure 1A,B). Additionally, miR‐425‐5p level in NMCFs was several times higher than that in NMCMs, indicating that miR‐425‐5p is primarily synthesized and distributed in NMCFs (Figure 1C).

Given that Ang II levels are elevated in HF patients and Ang II is commonly used to induce CMs hypertrophy and CFs activation in vitro, isolated NMCMs and NMCFs were then stimulated with Ang II. Intriguingly, after treatment with 1 μM Ang II, miR‐425‐5p expression in NMCFs was notably reduced in a time‐dependent manner, while no significant change of miR‐425‐5p level was observed in NMCMs treated with Ang II (Figure 1D,E). Thus, the decreased miR‐425‐5p levels in failing hearts and CFs, rather than in CMs, suggests that miR‐425‐5p mainly plays a functional role in fibroblast biology in HF pathogenesis. As a result, fibroblast‐specific overexpression of miR‐425‐5p was used in the subsequent experiments.

### 3.2 Specific overexpression of miR‐425‐5p in CFs attenuated cardiac dysfunction and hypertrophy induced by TAC in mice

**FIGURE 2:**
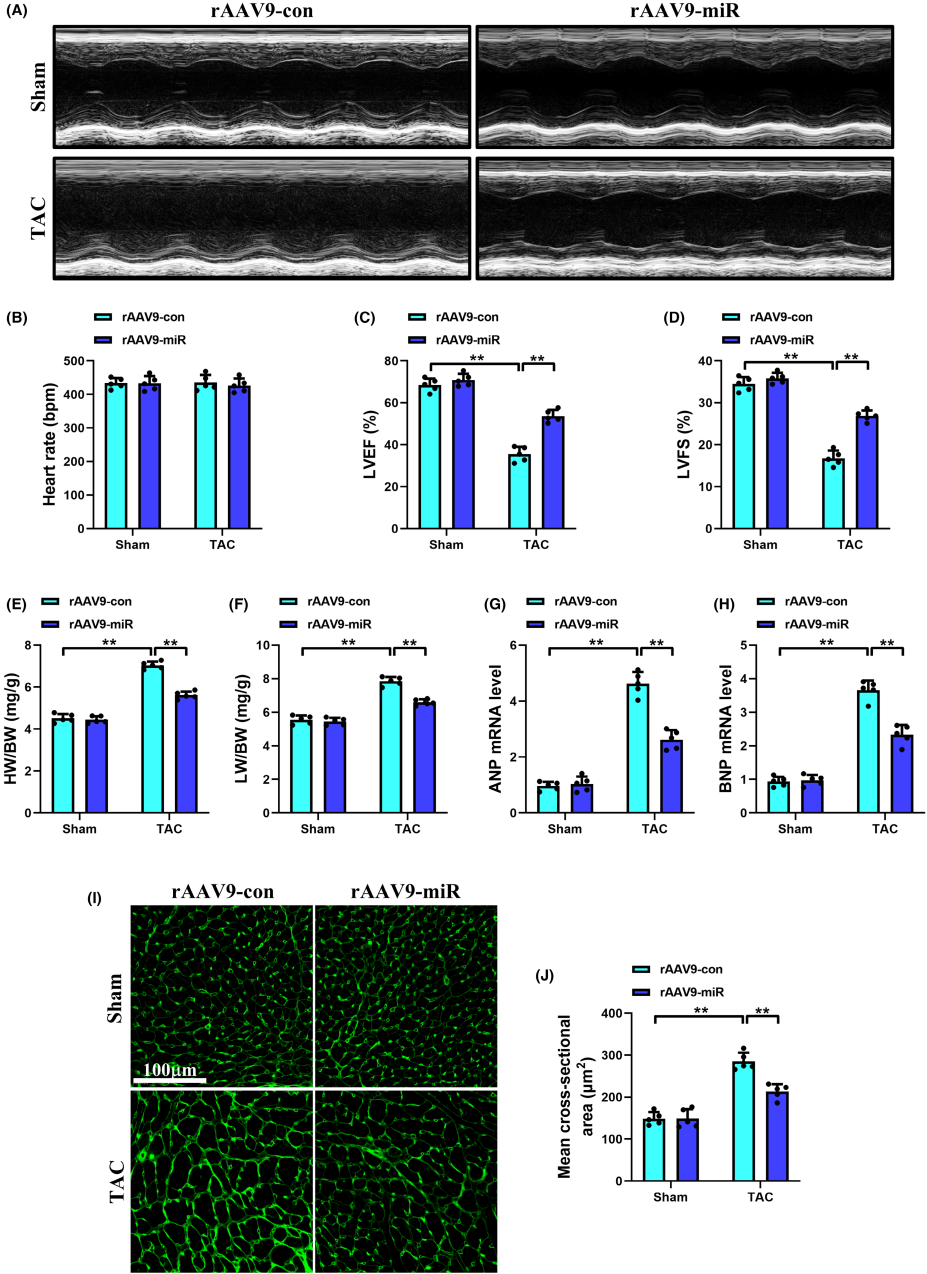
Specific overexpression of miR‐425‐5p in CFs attenuated cardiac dysfunction and hypertrophy induced by TAC in mice. (A) Representative M echocardiographic images of mice at 8 weeks after sham or TAC surgery. (B–D) Echocardiographic assessments of heart rate, LVEF and LVFS in each group (*n* = 5). (E, F) The ratios of HW/BW and LW/BW of mice at 8 weeks after sham or TAC surgery (*n* = 5). (G, H) Cardiac mRNA levels of hypertrophic markers ANP and BNP in each group (*n* = 5). (I) Representative images of WGA staining of heart tissues at 8 weeks after sham or TAC surgery (scale bar: 100 μm). (J) Quantification analysis of the mean CSA of cardiomyocyte in each group (*n* = 5). ANP, atrial natriuretic peptide; BNP, brain natriuretic peptide; CSA, cross‐sectional area; HW/BW, heart weight/body weight; LVEF, left ventricular ejection fraction; LVFS, left ventricular fractional shortening; LW/BW, lung weight/body weight; WGA, wheat germ agglutinin. Data were presented as mean ± SEM. Statistical significance was assessed by one‐way ANOVA. **p < 0.01.

Ultrasound echocardiography and measured parameters confirmed that cardiac systolic function in C57BL/6 mice was markedly impaired 8 weeks after TAC operation. As shown in Figure 2A–D, there was no significant difference in heart rate (HR) among groups, while LVEF and LVFS were strikingly decreased in the TAC + rAAV9‐con group compared with the Sham+rAAV9‐con group. Interestingly, rAAV9‐POSTN‐miR‐425‐5p reversed these impairments, as indicated by increased LVEF and LVFS values in the TAC + rAAV9‐miR group.

Eight weeks after TAC surgery, the HW/BW and LW/BW ratios in the TAC + rAAV9‐con group were remarkably higher than the corresponding ratios in the Sham+rAAV9‐con group. However, the phenomenon of TAC‐induced cardiac mass increase and lung edema was reversed by fibroblast‐specific overexpression of miR‐425‐5p (Figure 2E,F). Consistently, the levels of ANP and BNP, two classic markers of cardiac hypertrophy and HF, were significantly elevated in the TAC + rAAV9‐con group, which were also abrogated by rAAV9‐POSTN‐miR‐425‐5p administration in the TAC + rAAV9‐miR group (Figure 2G,H). Moreover, as shown in Figure 2I,J, WGA staining further confirmed the anti‐hypertrophic effects of miR‐425‐5p, as evidenced by the notable decrease in the CSA of CMs compared with the TAC + rAAV9‐con group. These findings indicate that fibroblast‐specific overexpression of miR‐425‐5p rescues TAC‐induced contractile dysfunction and cardiac hypertrophy.

### 3.3 Specific overexpression of miR‐425‐5p in CFs mitigated myocardial fibrosis and inhibited cardiac fibroblast activation induced by TAC in mice

**FIGURE 3:**
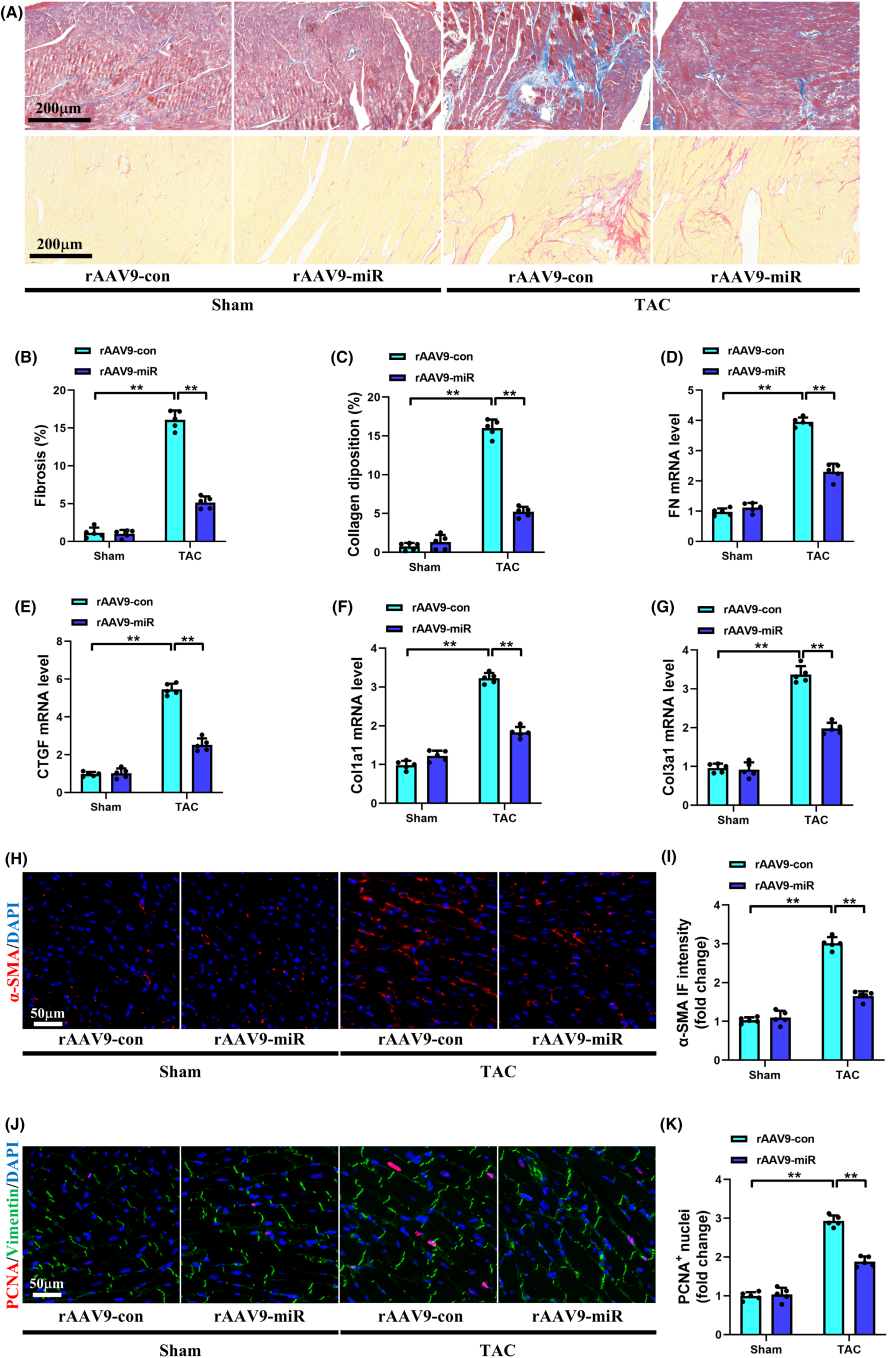
Specific overexpression of miR‐425‐5p in CFs mitigated myocardial fibrosis and inhibited cardiac fibroblasts activation induced by TAC in mice. (A) Representative images of Masson's trichrome and picric acid sirius red staining of heart tissues at 8 weeks after sham or TAC surgery (scale bar: 200 μm). (B, C) Quantification analyses of fibrosis area and collagen deposition in each group (*n* = 5). (D–G) Cardiac mRNA levels of fibrotic markers FN, CTGF, Col1a1 and Col3a1 in each group (*n* = 5). (H) Representative immunofluorescence images of α‐SMA (red) and DAPI (blue) stained heart tissues at 8 weeks after sham or TAC surgery (scale bar: 50 μm). (I) Semi‐quantitative analysis of α‐SMA immunofluorescence intensity in each group (*n* = 5). (J) Representative immunofluorescence images of PCNA (red), Vimentin (green) and DAPI (blue) stained heart tissues at 8 weeks after sham or TAC surgery (scale bar: 50 μm). (K) Semi‐quantitative analysis of PCNA^+^ nuclei in each group (*n* = 5). Col1a1, collagen 1a1; Col3a1, collagen 3a1; CTGF, connective tissue growth factor; FN, fibrotic markers fibronectin. Data were presented as mean ± SEM. Statistical significance was assessed by one‐way ANOVA. **p < 0.01.

Histological analysis showed that the impaired systolic function in TAC‐treated mice was associated with increased cardiac fibrosis. Masson's trichrome and picric acid sirius red staining revealed that, compared with the Sham+rAAV9‐con group, mice in the TAC + rAAV9‐con group exhibited dramatically augmented interstitial fibrosis and collagen deposition (Figure 3A–C). Meanwhile, transcriptional analysis of FN, CTGF, Col1a1 and Col3a1 further indicated that TAC surgery significantly promoted the expressions of these fibrotic markers (Figure 3D–G). However, the progression of myocardial fibrosis and the elevated levels of fibrotic markers were obviously reversed by rAAV9‐POSTN‐miR‐425‐5p administration in the TAC + rAAV9‐miR group.

Fibroblast differentiation and proliferation are key contributors to the progression of myocardial fibrosis. Immunofluorescent staining exhibited that the expression of α‐SMA, a marker of CFs transformation into myofibroblasts, was significantly increased in the TAC + rAAV9‐con group compared to the Sham+rAAV9‐con group (Figure 3H,I). Moreover, the number of PCNA and vimentin positive cells also exhibited the same trend following TAC operation, indicating the proliferation of CFs (Figure 3J,K). Similarly, the enhanced immunofluorescent intensities of α‐SMA and PCNA were attenuated after rAAV9‐POSTN‐miR‐425‐5p interference. Together, these findings suggest that fibroblast‐specific overexpression of miR‐425‐5p reverses pressure overload‐induced cardiac fibrosis by inhibiting CFs activation.

### 3.4 Overexpression of miR‐425‐5p inhibited cardiac fibroblast activation stimulated by Ang II in vitro

**FIGURE 4:**
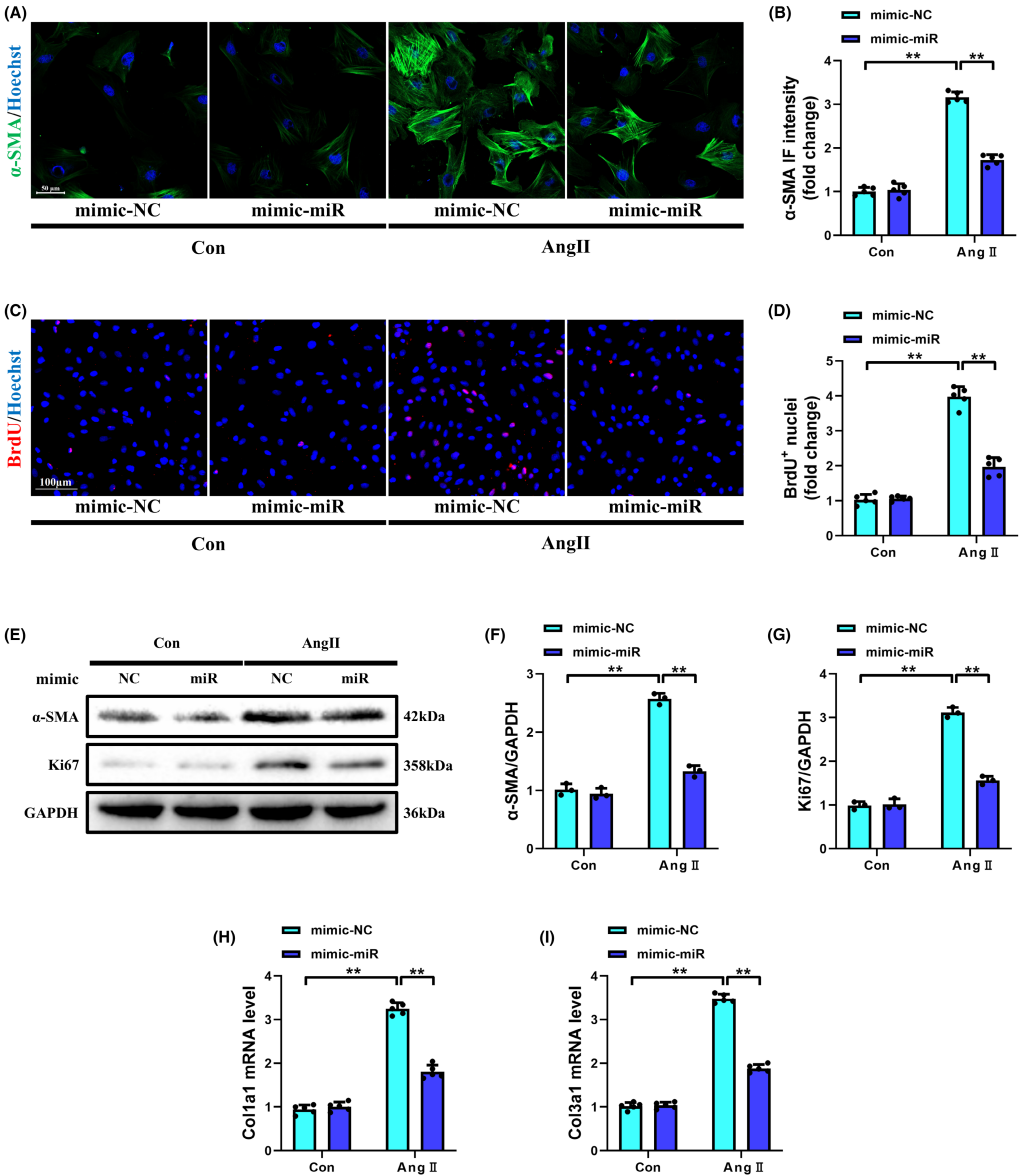
Overexpression of miR‐425‐5p inhibited cardiac fibroblasts activation stimulated by Ang II in vitro. (A) Representative immunofluorescence images of α‐SMA (green) and Hoechst (blue) stained NMCFs being treated with normal DMEM or Ang II for 24 h (scale bar: 50 μm). (B) Semi‐quantitative analysis of α‐SMA immunofluorescence intensity in each group (*n* = 5). (C) Representative immunofluorescence images of BrdU (red) and Hoechst (blue) stained NMCFs being treated with normal DMEM or Ang II for 24 h (scale bar: 100 μm). (D) Semi‐quantitative analysis of BrdU^+^ nuclei in each group (*n* = 5). (E) Representative immunoblots of α‐SMA, Ki67 and GAPDH (internal reference) of NMCFs being treated with normal DMEM or Ang II for 24 h. (F, G) Semi‐quantitative analyses of α‐SMA and Ki67 expressions in each group (*n* = 3). (H, I) Cellular mRNA levels of fibrotic markers Col1a1 and Col3a1 in each group (*n* = 5). Col1a1, collagen 1a1; Col3a1, collagen 3a1. Data were presented as mean ± SEM. Statistical significance was assessed by one‐way ANOVA. **p < 0.01.

In accordance with our in vivo results, miR‐425‐5p also inhibited the differentiation and proliferation of CFs in vitro. Immunostaining and Western blot analysis of α‐SMA were used to evaluate the transformation of CFs into myofibroblasts. Compared with the Con+mimic‐NC group, both the immunofluorescence intensity and protein expression of α‐SMA were significantly raised after 24 h of Ang II stimulation (Figure 4A,B,F). Simultaneously, cellular proliferation was assessed using IF assay of BrdU and Western blot detection of Ki67 in NMCFs (Figure 4C,D,G). Quantitative analysis showed that the number of BrdU‐positive CFs and Ki67 expression were markedly increased in the Ang II + mimic‐NC group compared to the Con+mimic‐NC group. In addition, the collagen synthesis capacity of NMCFs was evaluated through qRT‐PCR analysis of Col1a1 and Col3a1 transcription levels. Ang II‐induced upregulation of Col1a1 and Col3a1 mRNA expressions was abrogated when NMCFs were pre‐transfected with miR‐425‐5p mimics (Figure 4H,I). These data indicate that miR‐425‐5p inhibits Ang II‐induced CFs activation in vitro.

### 3.5 Overexpression of miR‐425‐5p inhibited the TGF‐β1/Smad signalling pathway both in vivo and in vitro

**FIGURE 5:**
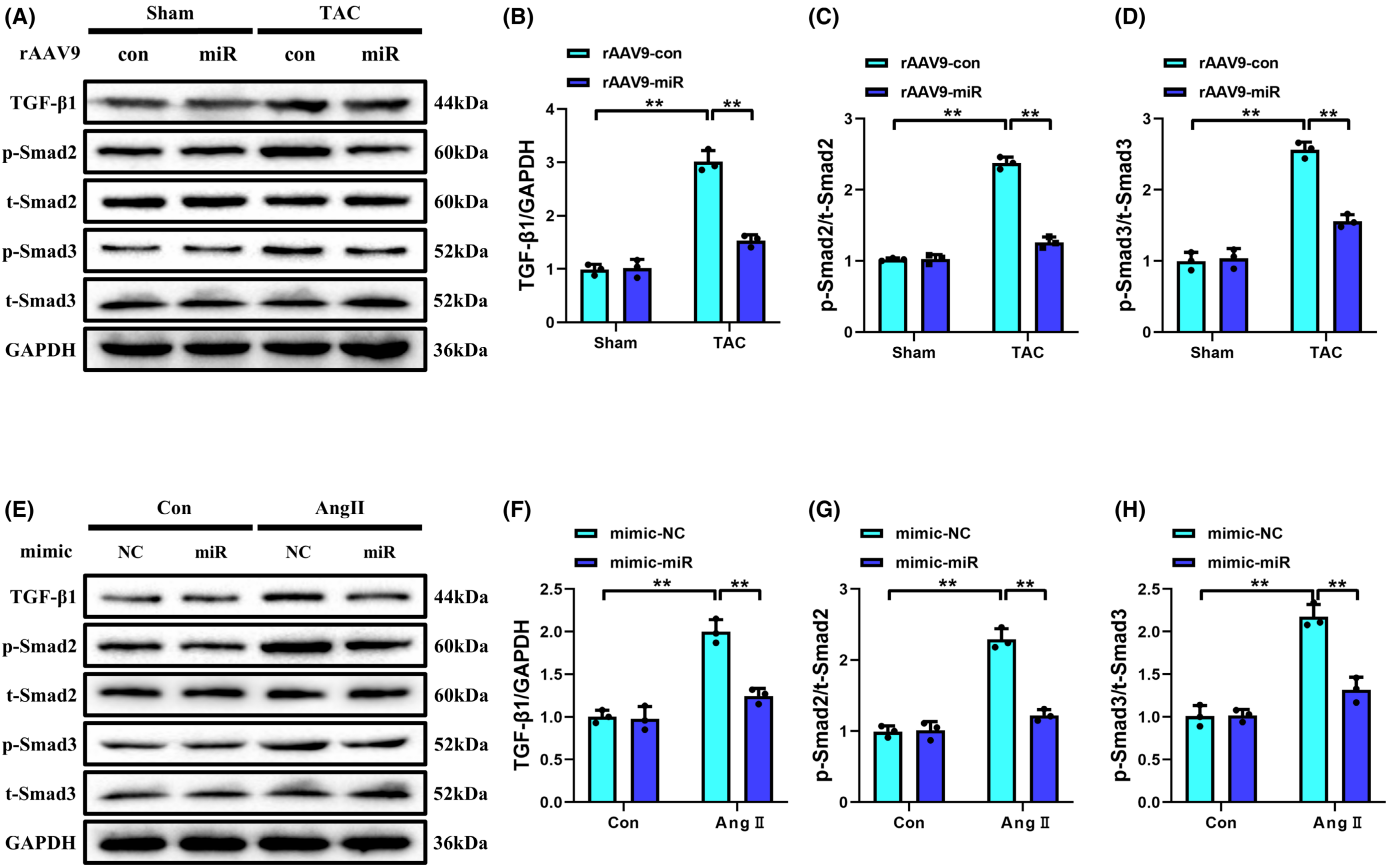
Overexpression of miR‐425‐5p inhibited the TGF‐β1/Smad signalling pathway both in vivo and in vitro. (A) Representative immunoblots of TGF‐β1, p‐Smad2, t‐Smad2, p‐Smad3, t‐Smad3 and GAPDH (internal reference) of heart tissues at 8 weeks after sham or TAC surgery. (B–D) Semi‐quantitative analyses of TGF‐β1, p‐Smad2 and p‐Smad3 expressions in each group (*n* = 3). (E) Representative immunoblots of TGF‐β1, p‐Smad2, t‐Smad2, p‐Smad3, t‐Smad3 and GAPDH (internal reference) of NMCFs being treated with normal DMEM or Ang II for 24 h. (F–H) Semi‐quantitative analyses of TGF‐β1, p‐Smad2 and p‐Smad3 expressions in each group (*n* = 3). Data were presented as mean ± SEM. Statistical significance was assessed by one‐way ANOVA. **p < 0.01.

TGF‐β1 is a major activator of CFs differentiation and proliferation, and it mediates the fibrotic response through several downstream fibrogenic pathways, particularly the Smad2/3 signalling. Thus, we examined TGF‐β1 expression and Smad2/3 phosphorylation to explore whether TGF‐β1/Smad signalling was involved in the antifibrotic effects of miR‐425‐5p. As shown in Figure 5A–D, the increased expressions of TGF‐β1 and p‐Smad2/3 induced by TAC were significantly reduced following rAAV9‐POSTN‐miR‐425‐5p administration, suggesting that the antifibrotic activity of miR‐425‐5p is closely associated with the suppression of TGF‐β1/Smad signalling.

Next, we verified the regulatory role of miR‐425‐5p on TGF‐β1/Smad signalling in vitro (Figure 5E–H). The results illustrated that compared with the Con+mimic‐NC group, TGF‐β1 expression and Smad2/3 phosphorylation increased sharply in NMCFs after incubation with Ang II for 24 h. However, Ang II‐induced activation of TGF‐β1/Smad signalling was remarkably blocked by miR‐425‐5p mimics, aligning with our in vivo results.

Considering that p‐Smad2/3 could directly bind to Smad4, forming a heterotrimeric complex that subsequently translocates into the nucleus to regulate the transcription of target genes, we also evaluated the translocation of Smad4. As shown in Figure S3, Smad4 significantly translocated from the cytoplasm to the nucleus in both in vivo and in vitro models, while the increased nuclear translocation of Smad4 was also markedly counteracted by miR‐425‐5p overexpression.

### 3.6 TGF‐β1/Smad signalling pathway was the downstream effector of miR‐425‐5p against Ang II‐stimulated cardiac fibroblast activation

**FIGURE 6:**
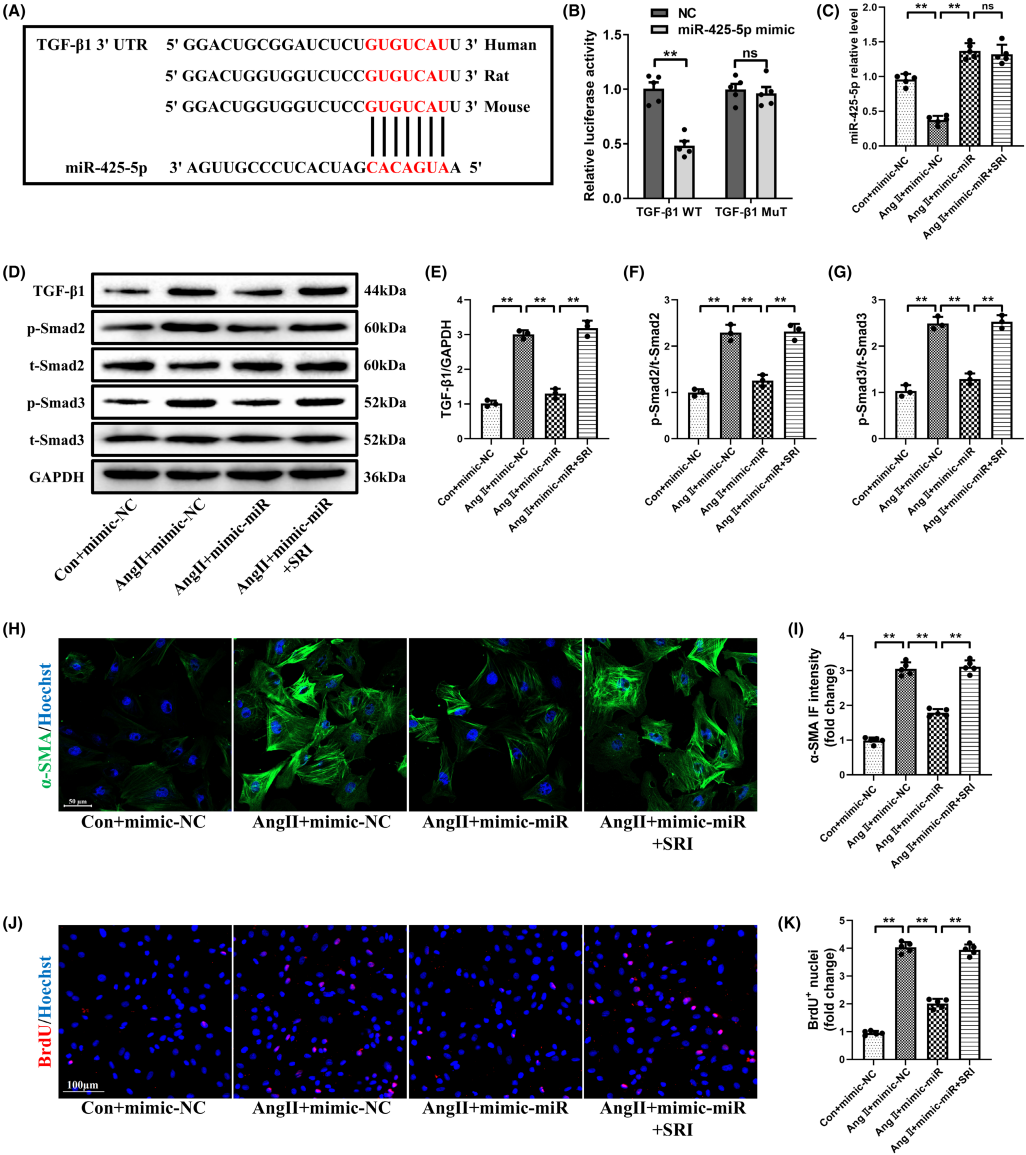
TGF‐β1/Smad signalling pathway was the downstream effector of miR‐425‐5p against Ang II‐stimulated cardiac fibroblast activation. (A) Bioinformatic analysis was performed to predict that TGF‐β1 was a potential target gene of miR‐425‐5p. (B) Luciferase assay was used to validate that miR‐425‐5p directly targeted TGF‐β1 (*n* = 5). (C) qPCR analysis of miR‐425‐5p level in NMCFs being treated with normal DMEM, Ang II or Ang II + SRI for 24 h (*n* = 5). (D) Representative immunoblots of TGF‐β1, p‐Smad2, t‐Smad2, p‐Smad3, t‐Smad3 and GAPDH (internal reference) of NMCFs being treated with normal DMEM, Ang II or Ang II + SRI for 24 h. (E–G) Semi‐quantitative analyses of TGF‐β1, p‐Smad2 and p‐Smad3 expressions in each group (*n* = 3). (H) Representative immunofluorescence images of α‐SMA (green) and Hoechst (blue) stained NMCFs being treated with normal DMEM, Ang II or Ang II + SRI for 24 h (scale bar: 50 μm). (I) Semi‐quantitative analysis of α‐SMA immunofluorescence intensity in each group (*n* = 5). (J) Representative immunofluorescence images of BrdU (red) and Hoechst (blue) stained NMCFs being treated with normal DMEM, Ang II or Ang II + SRI for 24 h (scale bar: 100 μm). (K) Semi‐quantitative analysis of BrdU^+^ nuclei in each group (*n* = 5). Data were presented as mean ± SEM. Statistical significance was assessed by one‐way ANOVA. **p < 0.01; NS, no significant.

To further prove that miR‐425‐5p ameliorated cardiac fibrosis via inhibiting the TGF‐β1/Smad pathway, we performed the luciferase reporter assay. Bioinformatic prediction indicated that miR‐425‐5p might directly bind the 3′UTR region of TGF‐β1 (Figure 6A). The luciferase reporter assay showed that miR‐425‐5p mimics induced a sharp decrease in the luciferase activity in HEK293 cells expressing the wild‐type sequence of TGF‐β1, but not in those expressing a mutant version (Figure 6B), supporting that miR‐425‐5p could directly target the 3′UTR of TGF‐β1 and suppress TGF‐β1 expression. This result is consistent with the findings from 3.5, where TGF‐β1 protein level was significantly reduced after miR‐425‐5p overexpression.

In addition, as shown in Figure 6C–G and Figure S3, utilizing SRI‐011381 (10 μM) to activate TGF‐β1 signalling resulted in substantial increases in TGF‐β1 expression, p‐Smad2/3 levels and Smad4 nuclear translocation, while having little impact on miR‐425‐5p expression. Furthermore, SRI‐011381 significantly abolished the inhibitory effects of miR‐425‐5p on Ang II‐stimulated CFs activation (Figure 6H–K). Taken together, these results indicate that TGF‐β1/Smad signalling is the key downstream effector of miR‐425‐5p in suppressing myocardial fibrosis.

### 3.7 Activation of TGF‐β1/Smad signalling abolished the protective effects of miR‐425‐5p against TAC‐induced cardiac dysfunction and hypertrophy

**FIGURE 7:**
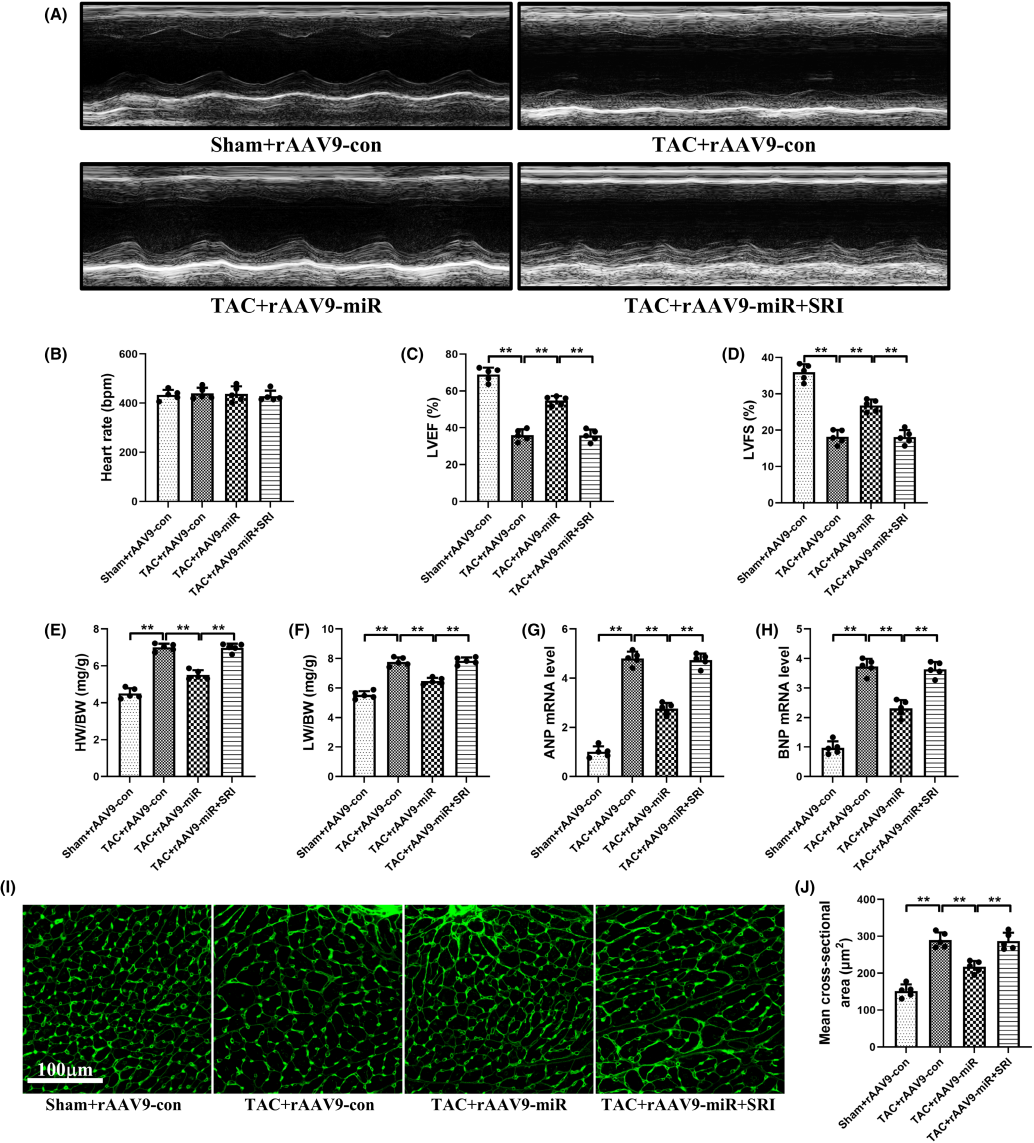
Activation of TGF‐β1/Smad signalling abolished the protective effects of miR‐425‐5p against TAC‐induced cardiac dysfunction and hypertrophy. (A) Representative M echocardiographic images of mice at 8 weeks after sham or TAC surgery. (B–D) Echocardiographic assessments of heart rate, LVEF and LVFS in the indicated groups. (E, F) The ratios of HW/BW and LW/BW of mice at 8 weeks after sham or TAC surgery (*n* = 5). (G, H) Cardiac mRNA levels of hypertrophic markers ANP and BNP in each group (*n* = 5). (I) Representative images of WGA staining of heart tissues at 8 weeks after sham or TAC surgery (scale bar: 100 μm). (J) Quantification analysis of the mean CSA of cardiomyocyte in each group (*n* = 5). ANP, atrial natriuretic peptide; BNP, brain natriuretic peptide; CSA, cross‐sectional area; HW/BW, heart weight/body weight; LVEF, left ventricular ejection fraction; LVFS, left ventricular fractional shortening; LW/BW, lung weight/body weight; WGA, wheat germ agglutinin. Data were presented as mean ± SEM. Statistical significance was assessed by one‐way ANOVA. **p < 0.01.

SRI‐011381 was intraperitoneally injected in order to verify that the TGF‐β1/Smad signalling pathway acts as a downstream effector of miR‐425‐5p in vivo. As shown in Figure 7A–D, compared with the TAC + rAAV9‐miR group, LVEF and LVFS were strikingly reduced in the TAC + rAAV9‐miR + SRI group. Besides, the HW/BW and LW/BW ratios in the TAC + rAAV9‐miR + SRI group were remarkably higher than those in the TAC + rAAV9‐miR group (Figure 7E,F). Consistently, the levels of ANP, BNP and the CSA of CMs were significantly elevated in the TAC + rAAV9‐miR + SRI group (Figure 7G–J).

### 3.8 Activation of TGF‐β1/Smad signalling abolished the protective effects of miR‐425‐5p against TAC‐induced myocardial fibrosis and CFs activation

**FIGURE 8:**
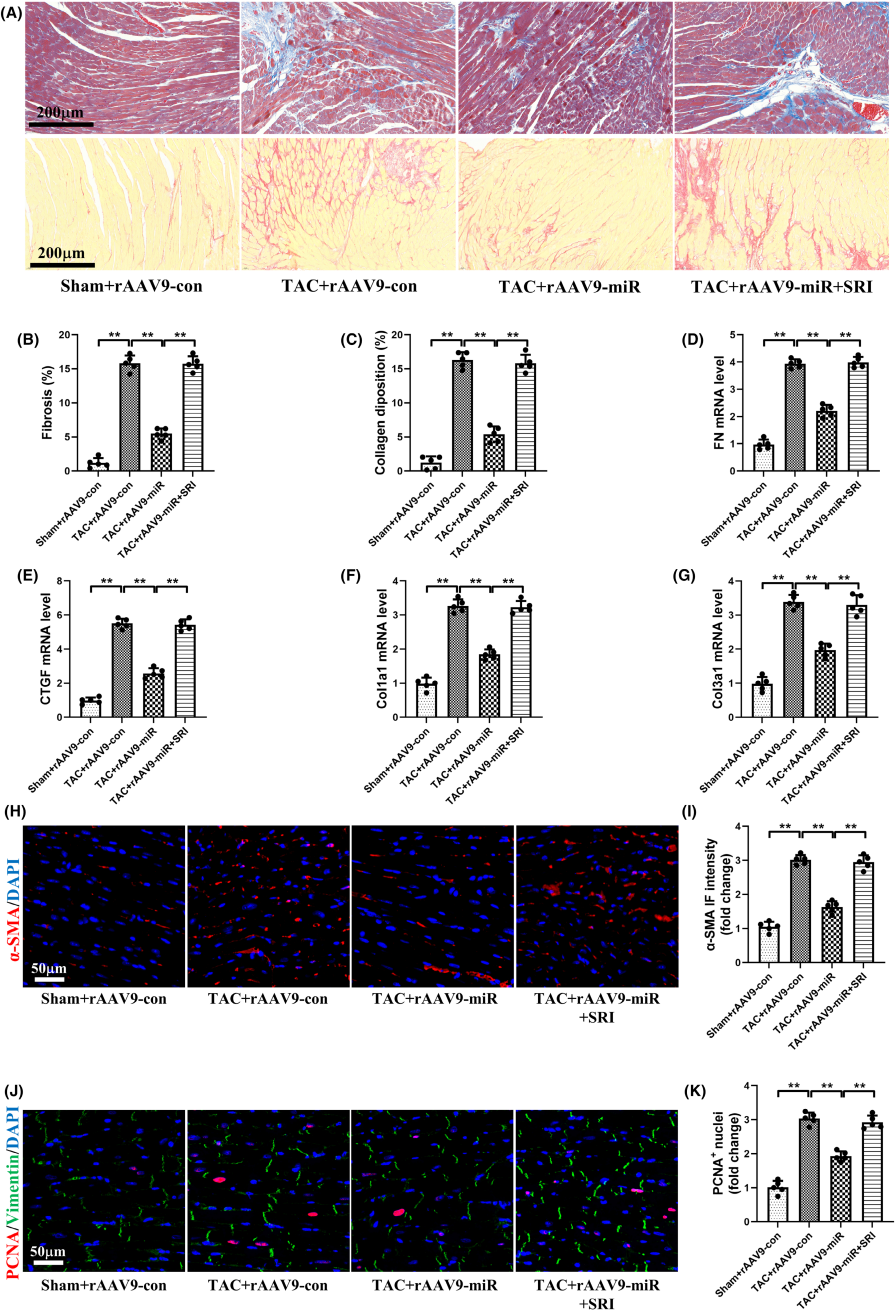
Activation of TGF‐β1/Smad signalling abolished the protective effects of miR‐425‐5p against TAC‐induced myocardial fibrosis and CFs activation. (A) Representative images of Masson's trichrome and picric acid sirius red staining of heart tissues at 8 weeks after sham or TAC surgery (scale bar: 200 μm). (B, C) Quantification analyses of fibrosis area and collagen deposition in each group (*n* = 5). (D–G) Cardiac mRNA levels of fibrotic markers FN, CTGF, Col1a1 and Col3a1 in each group (*n* = 5). (H) Representative immunofluorescence images of α‐SMA (red) and DAPI (blue) stained heart tissues at 8 weeks after sham or TAC surgery (scale bar: 50 μm). (I) Semi‐quantitative analysis of α‐SMA immunofluorescence intensity in each group (*n* = 5). (J) Representative immunofluorescence images of PCNA (red), Vimentin (green) and DAPI (blue) stained heart tissues at 8 weeks after sham or TAC surgery (scale bar: 50 μm). (K) Semi‐quantitative analysis of PCNA^+^ nuclei in each group (*n* = 5). Col1a1, collagen 1a1; Col3a1, collagen 3a1; CTGF, connective tissue growth factor; FN, fibrotic markers fibronectin. Data were presented as mean ± SEM. Statistical significance was assessed by one‐way ANOVA. **p < 0.01.

Masson's trichrome and picric acid sirius red staining revealed that mice in the TAC + rAAV9‐miR + SRI group exhibited dramatically augmented interstitial fibrosis and collagen deposition compared with the TAC + rAAV9‐miR group (Figure 8A–C). Meanwhile, the upregulated mRNA levels of FN, CTGF, Col1a1 and Col3a1 in the TAC + rAAV9‐miR + SRI group further indicated that SRI‐011381 administration significantly reversed the inhibitory effect of rAAV9‐POSTN‐miR‐425‐5p on myocardial fibrosis (Figure 8D–G).

Similarly, immunofluorescent staining exhibited that the expression of α‐SMA and the number of PCNA and vimentin positive cells were significantly increased in the TAC + rAAV9‐miR + SRI group compared with the TAC + rAAV9‐miR group (Figure 8H–K), suggesting that the activation of TGF‐β1/Smad signalling counteracted the inhibitory effect of rAAV9‐POSTN‐miR‐425‐5p on CFs proliferation and migration.

### 3.9 Supernatant from miR‐425‐5p‐trasfected CFs alleviated cardiomyocyte hypertrophy

**FIGURE 9:**
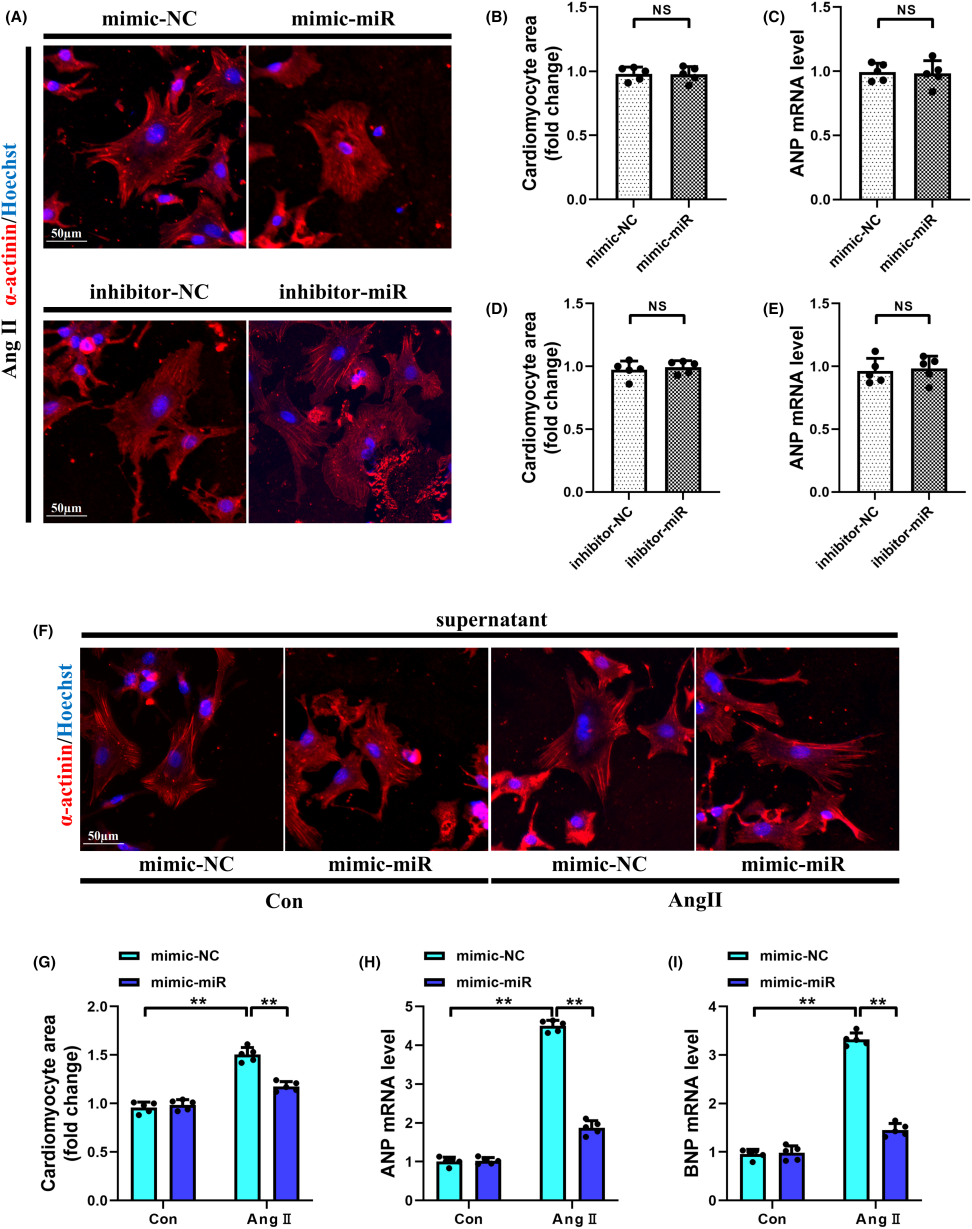
Supernatant from miR‐425‐5p‐trasfected CFs alleviated cardiomyocyte hypertrophy. (A) Representative immunofluorescence images of α‐actinin (red) and Hoechst (blue) stained NMCMs being treated with Ang II for 48 h (scale bar: 50 μm). (B) Quantification analysis of the mean CSA of NMCMs transfected with mimic‐NC or mimic‐miR (*n* = 5). (C) Cellular mRNA level of hypertrophic marker ANP in mimic‐treated NMCMs (*n* = 5). (D) Quantification analysis of the mean CSA of NMCMs transfected with inhibitor‐NC or inhibitor‐miR (*n* = 5). (E) Cellular mRNA level of hypertrophic marker ANP in inhibitor‐treated NMCMs (*n* = 5). (F) Representative immunofluorescence images of α‐actinin (red) and Hoechst (blue) stained NMCMs being treated with supernatant from miR‐425‐5p‐trasfected NMCFs for 48 h (scale bar: 50 μm). (G) Quantification analysis of the average CSA of NMCMs in each group (*n* = 5). (H, I) Cellular mRNA levels of hypertrophic markers ANP and BNP in each group (*n* = 5). ANP, atrial natriuretic peptide; BNP, brain natriuretic peptide; CSA, cross‐sectional area. Data were presented as mean ± SEM. Statistical significance was assessed by one‐way ANOVA. **p < 0.01; NS, no significant.

To clarify the role of miR‐425‐5p on cardiac hypertrophy, we initially silenced or overexpressed miR‐425‐5p in NMCMs. No significant changes in cell surface area or ANP expression were observed across different groups, indicating that altering the expression of miR‐425‐5p in NMCMs does not directly affect hypertrophy (Figure 9A–E).

Next, we used an indirect co‐culture method to examine whether the supernatant from activated CFs could affect CMs hypertrophy. We found that the supernatant from mimic‐NC treatment group led to an apparent increase in CMs surface area, accompanied by upregulated levels of ANP and BNP in CMs, whereas this pro‐hypertrophic effect was notably mitigated by the supernatant from miR‐425‐5p mimic‐treated CFs (Figure 9F–I).

## 4 DISCUSSION

The current study characterized the cell‐type‐specific role of miR‐425‐5p in TAC‐induced HF and further identified the molecular mechanisms responsible. Specifically, we found that overexpression of miR‐425‐5p in CFs ameliorated cardiac hypertrophy, mitigated myocardial fibrosis and subsequently improved cardiac function after TAC surgery. Mechanistically, upregulation of miR‐425‐5p regulated cardiac remodelling by inhibiting CFs transformation and differentiation, as well as suppressing collagen synthesis and deposition through directly targeting the TGF‐β1/Smad signalling. The present experimental results indicate that overexpression of fibroblast‐derived miR‐425‐5p plays a protective role in curbing the progression of cardiac remodelling and retarding the transition from pathological hypertrophy to HF.

HF is a clinical disorder caused by cardiac overload or irreversible myocardial damage, leading to significant morbidity and mortality worldwide.^1^ The discovery that several miRNAs are differentially expressed in HF cohorts has suggested their possible involvement in the pathogenesis of HF.^26^, ^27^ Among these miRNAs, miR‐425 is a recently studied one that exerts protective functions against multiple CVDs.^12^, ^13^, ^14^, ^15^ In particular, it has been reported that plasma concentrations of miR‐425 are declined in failing hearts, and the level of miR‐425 is closely correlated with ANP production, suggesting miR‐425 could be a non‐invasive clinical biomarker for HF.^16^, ^28^, ^29^ Similarly, the present research noted that miR‐425‐5p levels were remarkably downregulated in both the global heart tissue and plasma in TAC‐caused HF mice, implying that miR‐425‐5p may also participate in the modulation of pressure overload‐induced HF.

Myocardial fibrosis plays a critical role in the evolution of adverse cardiac remodelling and the development of HF in patients with CVDs.^8^ Interstitial fibrosis, which promotes myocardial stiffness and decreases cardiac compliance, ultimately leads to the progression of cardiac dysfunction.^30^ Currently, miR‐425 has been described to be implicated in the course of fibrosis response.^31^, ^32^ More importantly, Wang et al. reported that reduced miR‐425 levels contribute to the pathogenesis of myocardial fibrosis, and changes in miR‐425 levels modulate the expression of fibrosis‐related target genes.^16^ Wei et al. demonstrated that miR‐425‐5p could serve as a novel biomarker for atrial fibrosis and contribute to atrial remodelling in atrial fibrillation.^17^ Given the potential role of miR‐425‐5p in myocardial fibrosis, we aimed to ascertain its functions during this process in the model of TAC‐induced HF.

It is universally acknowledged that CFs are not only the predominant cardiac cells in normal hearts but also the primary effector cells in cardiac remodelling.^33^ In fact, excessive proliferation of CFs and their differentiation into myofibroblasts have been identified as key mechanisms driving the fibrotic process.^34^, ^35^ Additionally, Ang II, the principal mediator of the renin‐angiotensin system, functions as a potent pro‐fibrotic molecule in various pathological states.^36^, ^37^ By incubating isolated NMCFs and NMCMs with Ang II, we first found that miR‐425‐5p expression was notably declined in NMCFs in a time‐dependent manner, but not significantly in NMCMs, suggesting that miR‐425‐5p might mainly play a functional role in fibroblast biology under pressure‐overload stimuli. Then, rAAV9 was employed to overexpress miR‐425‐5p specifically in CFs, enabling us to further explore the cell‐specific effects of miR‐425‐5p in the development of HF. Our data showed that fibroblast‐specific overexpression of miR‐425‐5p markedly reversed TAC‐induced proliferation, secretion and transformation of CFs, which in turn mitigated myocardial fibrosis and collagen deposition, ultimately improving cardiac function. Moreover, consistent with the in vivo results, our in vitro experiments further confirmed the anti‐fibrotic actions of miR‐425‐5p in inhibiting CFs activation and weakening their matrix‐synthetic capacity at the cellular level. These data clearly indicate that fibroblast‐specific enhancement of miR‐425‐5p could indeed exert protective effects against HF by suppressing myocardial fibrosis.

It is well‐known that CFs are a major source of TGF‐β, which, in turn, regulates the proliferation and differentiation of CFs.^38^, ^39^ Among the several isoforms of TGF‐β, TGF‐β1 is the most abundant and commonly expressed.^40^ Growing data confirmed that myocardial TGF‐β1 synthesis is markedly and consistently upregulated in animal models of HF,^41^ and overexpression of TGF‐β1 accelerates the progression of myocardial fibrosis via the canonical Smad2/3 signalling.^42^, ^43^ Notably, there is evidence that miR‐425 could modulate the TGF‐β family pathway. Du et al. revealed that miR‐425 enhanced granulosa cells apoptosis by targeting the canonical TGF‐β signalling pathway.^19^ Liu et al. concluded that miR‐425 suppressed the development of triple‐negative breast cancer by targeting the TGF‐β1/Smad3 signalling pathway.^20^ Furthermore, miR‐425‐5p has been found to play a critical role in inhibiting myofibroblast differentiation by reducing the TGF‐β1 expression in dermal fibroblasts.^44^ Most importantly, overexpressed miR‐425‐3p was found to inhibit cardiomyocyte apoptosis and myocardial inflammation by suppressing the TGF‐β1/Smad axis.^13^ However, whether the TGF‐β1/Smad signalling could mediate the anti‐fibrotic effects of miR‐425‐5p in pressure overload‐caused HF remains to be elucidated. Results of our study found that TAC or Ang II markedly upregulated TGF‐β1 expression and Smad2/3 phosphorylation, both of which were abrogated by overexpression of miR‐425‐5p in CFs. A luciferase activity assay verified that miR‐425‐5p could directly bind to TGF‐β1. Besides, SRI‐011381, an activator of TGF‐β signalling, reversed the inhibitory action of miR‐425‐5p on TAC‐ or Ang II‐induced CFs activation, without affecting miR‐425‐5p levels. These results indicate that TGF‐β1/Smad pathway might be the direct downstream target of miR‐425‐5p in alleviating myocardial fibrosis.

Interestingly, our experiments also observed that TAC‐induced cardiac hypertrophy, another key aspect of cardiac remodelling, was prominently alleviated by fibroblast‐specific upregulation of miR‐425‐5p. Since no significant changes in miR‐425‐5p levels were seen in NMCMs treated with Ang II, and neither inhibition nor overexpression of miR‐425‐5p in CMs had a significant effect on Ang II‐induced cell hypertrophy, we hypothesized that the anti‐hypertrophic action of CFs‐derived miR‐425‐5p may be mediated via intercellular interaction.

Given the close proximity between CFs and CMs, their communication is critical for maintaining cardiac homeostasis under physiologic conditions and promoting cardiac repair in pathological states.^45^, ^46^ Numerous studies have indicated that the complex crosstalk between CMs and CFs can influence each other's function in the pathophysiology of cardiac remodelling. On one hand, damaged CMs could activate CFs through multiple signalling molecules, such as reactive oxygen species (ROS), TGF‐β1 and Ang II, via paracrine action, thereby promoting myocardial fibrosis.^47^ On the other hand, the transformation of fibroblasts into myofibroblasts during myocardial injury or chronic stress is accompanied by the release of many biologically active molecules that amplify the hypertrophic response in CMs.^48^ Likewise, our in vitro findings demonstrated that the supernatant from miR‐425‐5p‐trasfected CFs significantly alleviated CMs hypertrophy, suggesting that miR‐425‐5p may indirectly participate in the fibroblast‐to‐cardiomyocyte crosstalk by inhibiting the secretion of pro‐hypertrophic mediators from activated CFs. However, the precise mechanism by which CFs‐derived miR‐425‐5p regulates CMs biology remains unknown, representing a limitation of this study and warranting further investigation.

## 5 CONCLUSION

In summary, using a TAC‐induced mouse model and an Ang II‐treated NMCFs model, we found that the overexpression of miR‐425‐5p in CFs significantly ameliorated cardiac remodelling and contractile dysfunction during the pathological progression of HF. Specifically, the anti‐fibrotic actions of miR‐425‐5p were achieved by inhibiting CFs activation through the suppression of the TGF‐β1/Smad signalling. Additionally, miR‐425‐5p may confer protection against cardiac hypertrophy via fibroblast‐cardiomyocyte crosstalk. This research highlights the protective role of fibroblast‐derived miR‐425‐5p in pressure overload‐induced HF and suggests that miR‐425‐5p could serve as a promising diagnostic biomarker and a therapeutic target for preventing and treating adverse remodelling in HF patients.

## AUTHOR CONTRIBUTIONS

**Haijia Zhou:** Methodology (lead); writing – original draft (lead); writing – review and editing (equal). **Pengyun Liu:** Writing – original draft (equal); writing – review and editing (equal). **Xuelin Guo:** Data curation (lead); methodology (equal); supervision (equal). **Wei Fang:** Methodology (equal); software (lead); supervision (equal). **Chan Wu:** Supervision (equal); writing – review and editing (equal). **Mingming Zhang:** Conceptualization (equal); funding acquisition (lead); supervision (equal). **Zhaole Ji:** Conceptualization (lead); project administration (lead); supervision (lead).

## FUNDING INFORMATION

This study was financially supported by the National Natural Science Foundation of China [grant numbers 82270366].

## CONFLICT OF INTEREST STATEMENT

The authors declare that they have no known competing financial interests or personal relationships that could have appeared to influence the work reported in this paper.

## Supporting information


**Figure S1:** Protocol of in vivo experiments ①.

**Figure S2:** Protocol of in vivo experiments ②.

**Figure S3:** The expression of Nuclear Smad4 in each group. (A) Left, representative immunoblots of Nuclear Smad4 and Histone H3 (internal reference) of heart tissues at 8 weeks after sham or TAC surgery. Right, Semi‐quantitative analysis of Nuclear Smad4 expression in each group (*n* = 3). (B) Left, representative immunoblots of Nuclear Smad4 and Histone H3 (internal reference) of NMCFs being treated with normal DMEM or Ang II for 24 h. Right, Semi‐quantitative analysis of Nuclear Smad4 expression in each group (*n* = 3). (C) Left, representative immunoblots of Nuclear Smad4 and Histone H3 (internal reference) of NMCFs being treated with normal DMEM, Ang II or Ang II + SRI for 24 h. Right, Semi‐quantitative analysis of Nuclear Smad4 expression in each group (*n* = 3). **p < 0.01.

**Figure S4:** Detection of rAAV9 infection efficiency. (A) Representative images of green fluorescent protein in heart tissues at 4 weeks after tail vein injection of rAAV9‐POSTN‐con or rAAV9‐POSTN‐miR‐425‐5p (scale bar: 10 μm). (B) qPCR analysis of miR‐425‐5p level in the heart tissues at 4 weeks after tail vein injection of rAAV9‐POSTN‐con or rAAV9‐POSTN‐miR‐425‐5p (*n* = 5). **p < 0.01.

**Figure S5:** The concentration of SRI‐011381 used in this study had no obvious toxicity to mice and NMCFs. (A, B) Echocardiographic assessments of LVEF and LVFS in the indicated groups (*n* = 5). (C) Comparison of cell viability between the Con group and Con+SRI group (*n* = 5). NS, no significant.

## Data Availability

The data that support the findings of this study are available from the corresponding author upon reasonable request.
