# Supplementary figures and images for: Fibroblast‐derived miR‐425‐5p alleviates cardiac remodelling in heart failure via inhibiting the TGF‐β1/Smad signalling
Source: J Cell Mol Med. 2024 Nov 11;28(21):e70199. doi: 10.1111/jcmm.70199 (PMC11552651; doi:10.1111/jcmm.70199)

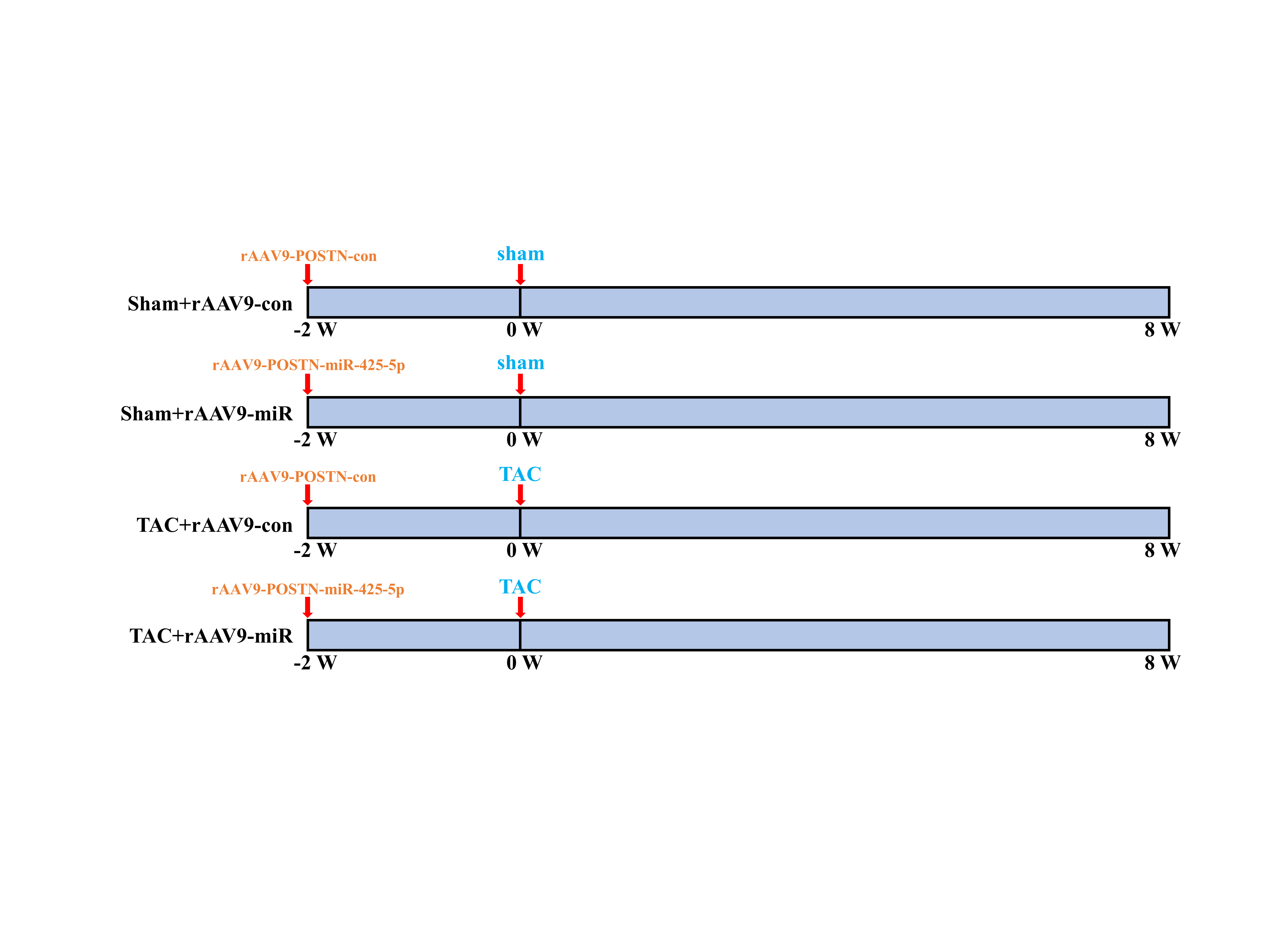

Supplement: Supplementary file 1 — Figure S1: Protocol of in vivo experiments ①. [file JCMM-28-e70199-s005.tif]

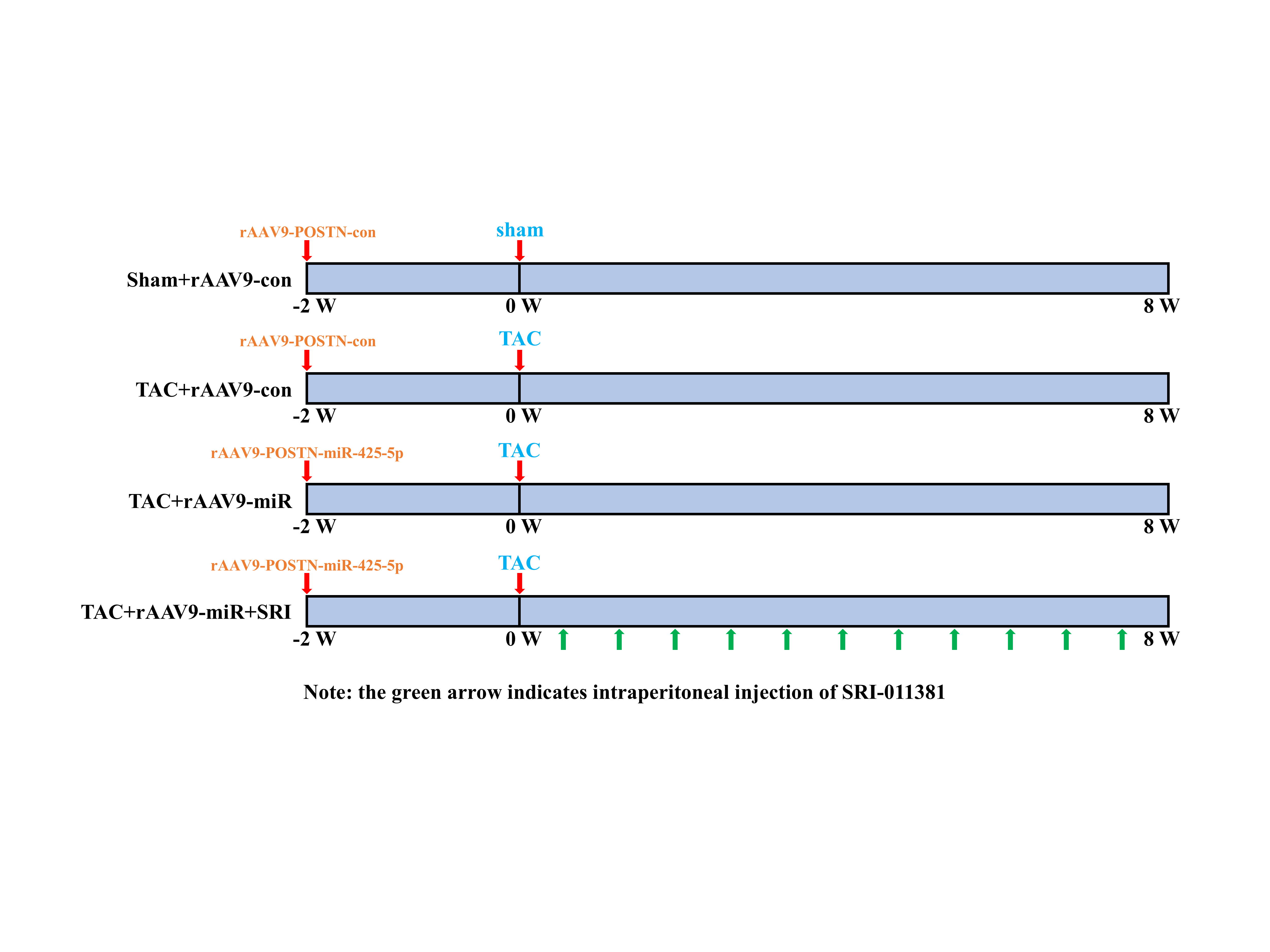

Supplement: Supplementary file 2 — Figure S2: Protocol of in vivo experiments ②. [file JCMM-28-e70199-s002.tif]

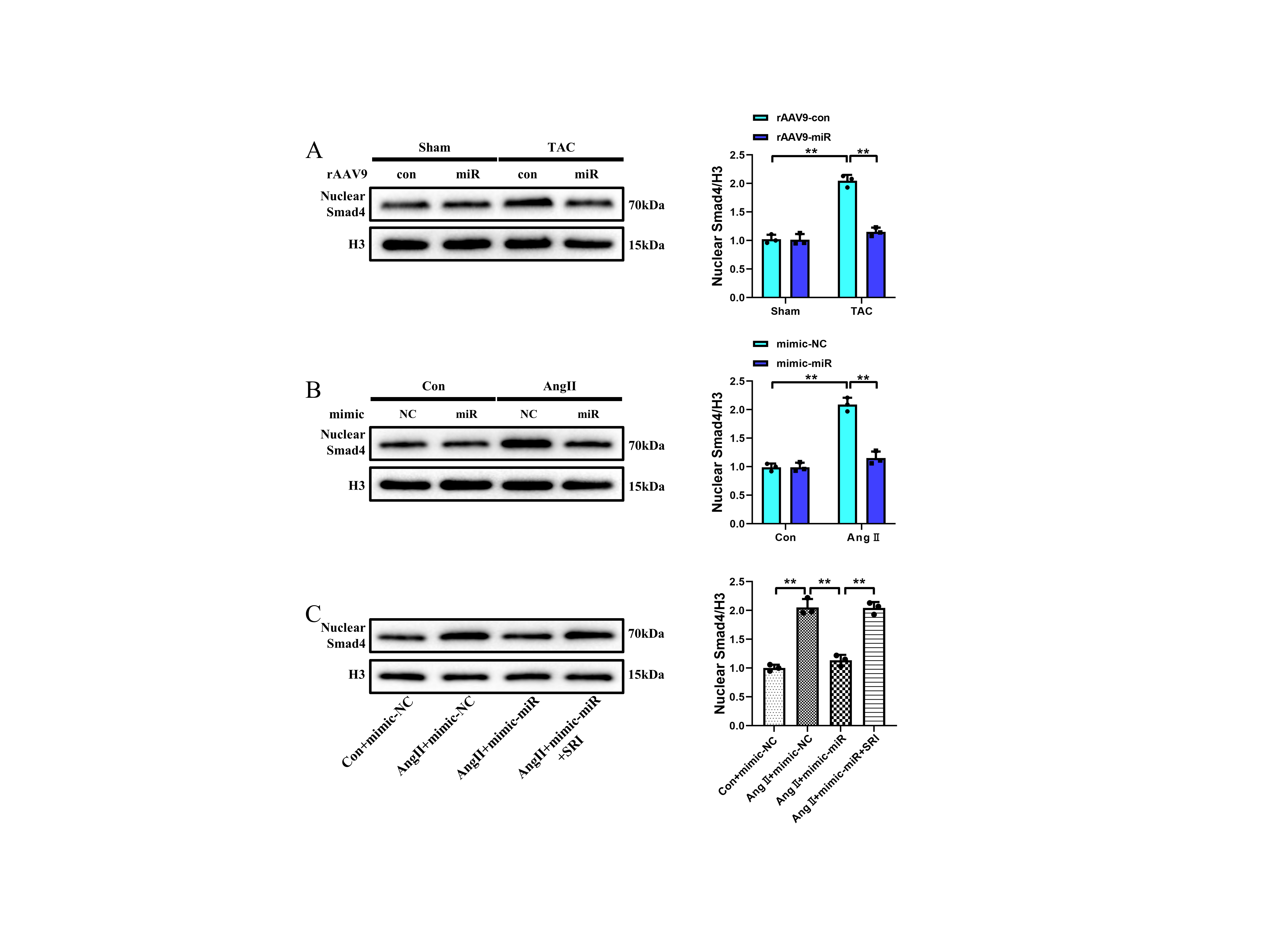

Supplement: Supplementary file 3 — Figure S3: The expression of Nuclear Smad4 in each group. (A) Left, representative immunoblots of Nuclear Smad4 and Histone H3 (internal reference) of heart tissues at 8 weeks after sham or TAC surgery. Right, Semi‐quantitative analysis of Nuclear Smad4 expression in each group (n = 3). (B) Left, representative immunoblots of Nuclear Smad4 and Histone H3 (internal reference) of NMCFs being treated with normal DMEM or Ang II for 24 h. Right, Semi‐quantitative analysis of Nuclear Smad4 expression in each group (n = 3). (C) Left, representative immunoblots of Nuclear Smad4 and Histone H3 (internal reference) of NMCFs being treated with normal DMEM, Ang II or Ang II + SRI for 24 h. Right, Semi‐quantitative analysis of Nuclear Smad4 expression in each group (n = 3). **p < 0.01. [file JCMM-28-e70199-s004.tif]

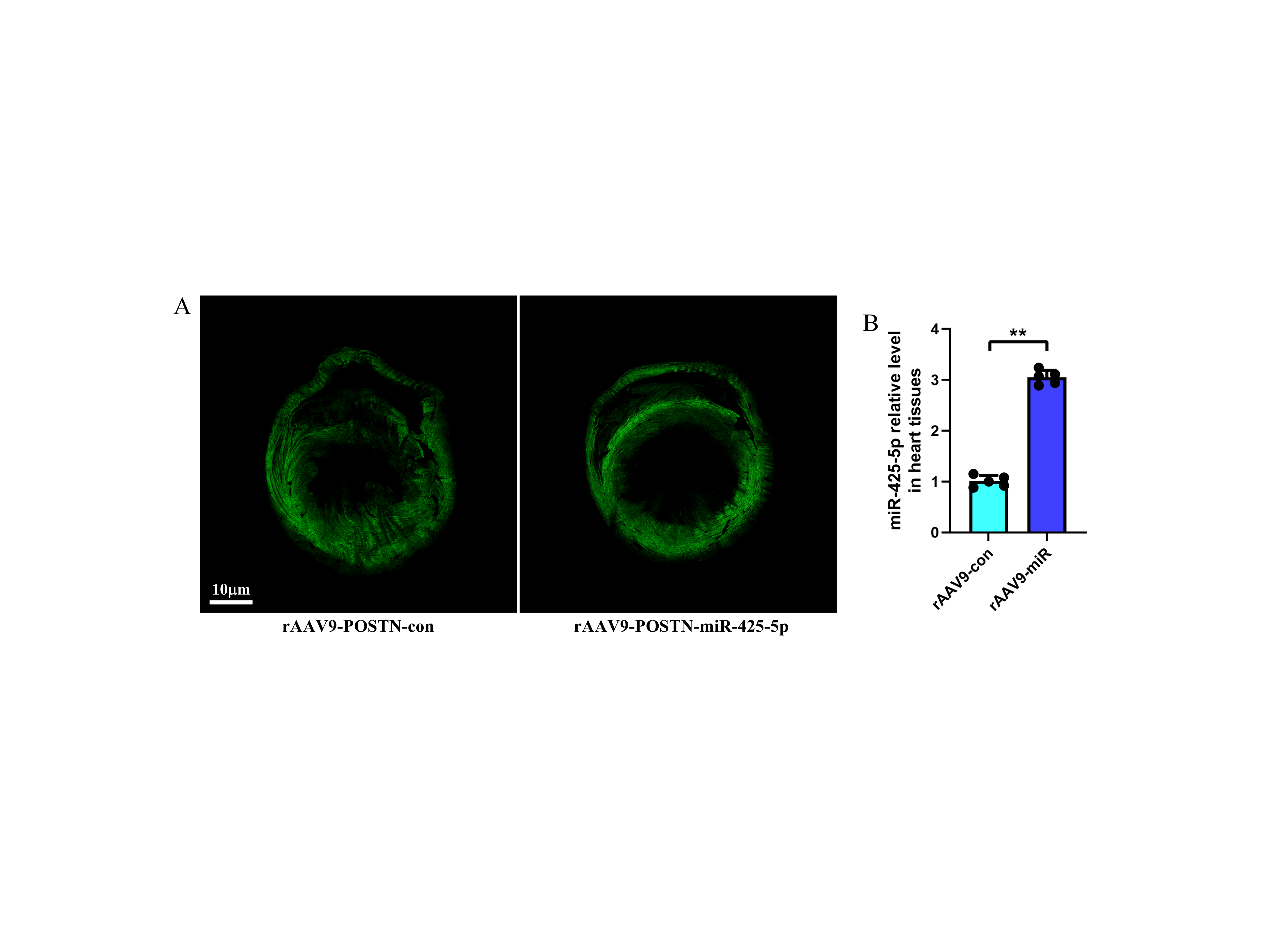

Supplement: Supplementary file 4 — Figure S4: Detection of rAAV9 infection efficiency. (A) Representative images of green fluorescent protein in heart tissues at 4 weeks after tail vein injection of rAAV9‐POSTN‐con or rAAV9‐POSTN‐miR‐425‐5p (scale bar: 10 μm). (B) qPCR analysis of miR‐425‐5p level in the heart tissues at 4 weeks after tail vein injection of rAAV9‐POSTN‐con or rAAV9‐POSTN‐miR‐425‐5p (n = 5). **p < 0.01. [file JCMM-28-e70199-s001.tif]

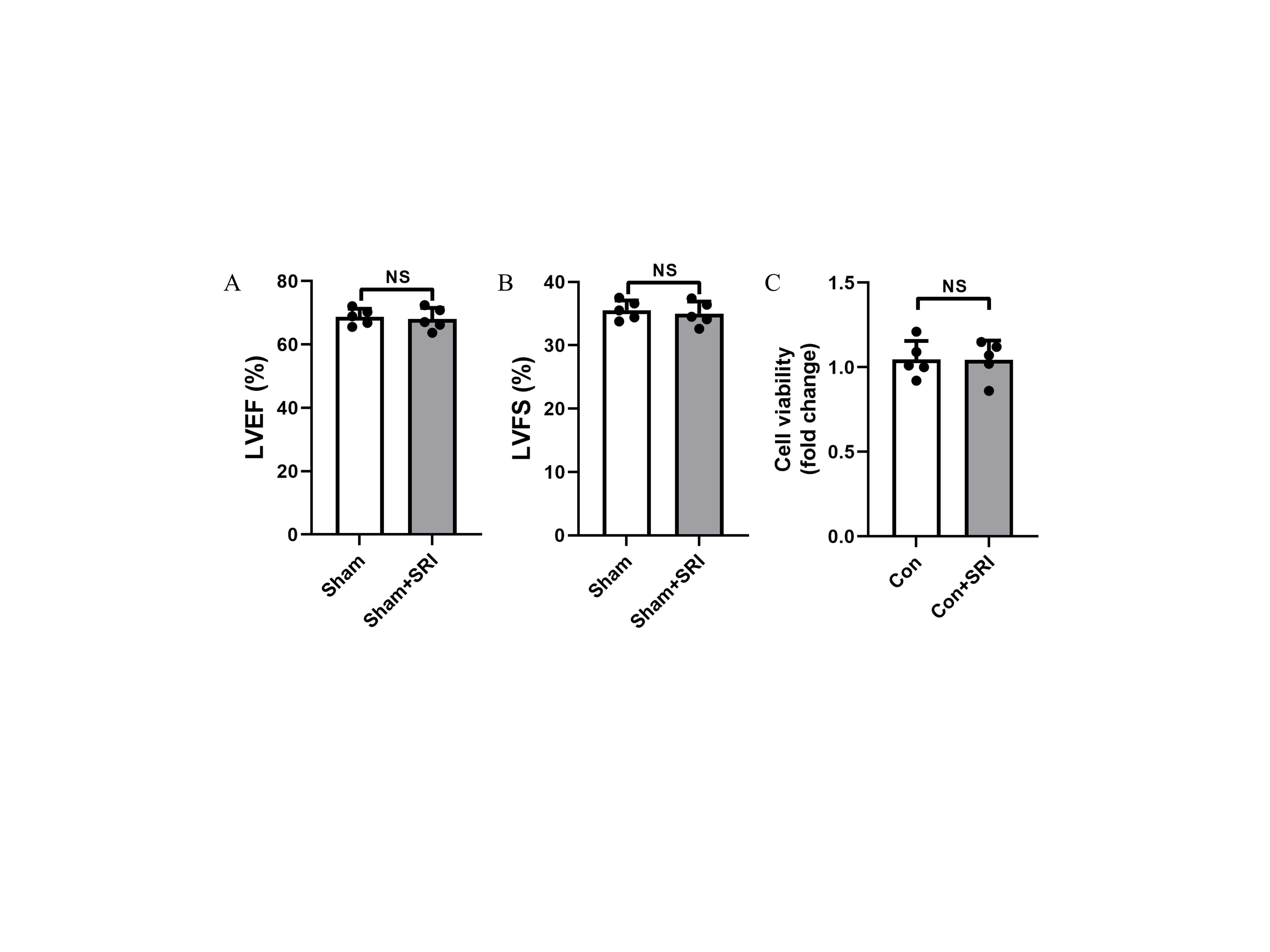

Supplement: Supplementary file 5 — Figure S5: The concentration of SRI‐011381 used in this study had no obvious toxicity to mice and NMCFs. (A, B) Echocardiographic assessments of LVEF and LVFS in the indicated groups (n = 5). (C) Comparison of cell viability between the Con group and Con+SRI group (n = 5). NS, no significant. [file JCMM-28-e70199-s003.tif]
